# Integrative studies on the taxonomy and molecular phylogeny of four new *Pleuronema* species (Protozoa, Ciliophora, Scuticociliatia)

**DOI:** 10.1007/s42995-022-00130-5

**Published:** 2022-05-12

**Authors:** Mingjian Liu, Yujie Liu, Tengteng Zhang, Borong Lu, Feng Gao, Jing Gu, Saleh A. Al-Farraj, Xiaozhong Hu, Weibo Song

**Affiliations:** 1 Institute of Evolution and Marine Biodiversity, Ocean University of China, Qingdao, 266003 China; 2 College of Fisheries, Ocean University of China, Qingdao, 266003 China; 3 Laboratory of Protozoological Biodiversity and Evolution in Wetland, College of Life Sciences, Shaanxi Normal University, Xi’an, 710119 China; 4 Qingdao No. 47 Middle School, Qingdao, 266022 China; 5 Zoology Department, College of Science, King Saud University, Riyadh, 11451 Saudi Arabia; 6 Laboratory for Marine Biology and Biotechnology, Pilot National Laboratory for Marine Science and Technology (Qingdao), Qingdao, 266237 China

**Keywords:** Ciliates, Ciliature, Morphology, New species, Pleuronematids, SSU rRNA gene

## Abstract

The ciliate genus *Pleuronema* comprises approximately 30 nominal species and has been reported in freshwater, brackish water, and marine habitats. Nevertheless, recent studies have indicated that there might be a large undiscovered species diversity. In the present work, four new *Pleuronema* species, namely *P. foissneri* sp. nov., *P. parasmalli* sp. nov., *P. parasalmastra* sp. nov., and *P. paraorientale* sp. nov., collected from Shenzhen, southern China, was investigated using taxonomic methods. The diagnosis, description, comparisons with morphologically related species and detailed morphometric data are supplied for each. The small subunit ribosomal RNA (SSU rRNA) gene of the four new species is sequenced and their molecular phylogeny is analyzed. The SSU rRNA gene tree shows that *Pleuronema* is polyphyletic comprising several separate clades. All four new species cluster consistently with *P. orientale* KF206429, *P. puytoraci* KF840520 and *P. setigerum* FJ848874 within the core Pleuronematidae + Peniculistomatidae clade. Phylogenies of Pleuronematidae-related taxa are also discussed.

**Supplementary Information:**

The online version contains supplementary material available at 10.1007/s42995-022-00130-5.

## Introduction

The ciliate genus *Pleuronema* was established by Dujardin (1841), based on its ovoidal body with a depression, long oral opening, and prominent oral cilia, in a redescription of “*Paramecium chrysalis*” by Ehrenberg (1838), which is very distinct from other reports on *Paramecium*. Dujardin (1841) also described another two *Pleuronema* species: *P. crassa* and *P. marina*. Today, *Pleuronema* is a speciose and cosmopolitan genus comprising approximately 30 nominal species which can be found in various aquatic environments (Fan and Pan 2020; Hu et al. 2019; Lynn 2008; Pan et al. 2015a, 2016; Song et al. 2009). In recent years, several new species have been discovered, suggesting there is a large undescribed diversity and a need to conduct further studies on this genus (Pan et al. 2015a, b, 2016; Wang et al. 2008a, b, 2009). Molecular phylogenetic analyses have increasingly been applied in modern taxonomic studies on ciliated protists, and the polyphyly of *Pleuronema* has been widely reported (Antipa et al. 2016, 2020; Gao et al. 2012, 2013, 2017; Pan et al. 2010, 2015a, b, 2016, 2020; Yi et al. 2009).

Although *Pleuronema* is a common genus with a long research history, there are still problems with its taxonomy. These include: (1) Incomplete morphological information: some species, especially those described in the eighteenth and nineteenth centuries, lack detailed descriptions or graphic illustrations. For example, eight species lack illustrations or photomicrographs of cells in vivo and the whole ciliature pattern remains unknown for *P. grassei*, *P. prunulum*, and *P. simplex*. Consequently, these poorly known taxa have not been reported since they were first described (Agatha et al. 1993; Corliss and Snyder 1986; Dragesco 1960, 1968; Fernandez-Leborans and Novillo 1994; Kahl 1926). (2) Confusion in taxonomic status: some characters from live observation (e.g., right ventrolateral side straight or convex) and silver-stained specimens (e.g., position and length of the buccal field relative to the body length, anterior position of membranelle 2b) were overlooked until the middle of the twentieth century causing unreliable identifications and synonyms/homonyms (Agamaliev 1968; Borror 1963, 1972; Dragesco 1960, 1968; Noland 1937). (3) Insufficient molecular data: SSU rRNA gene sequence data are available for only half of nominal *Pleuronema* species and a large proportion of these sequences lack corresponding morphological information, thus their identity needs to be confirmed (Gao et al. 2013). In addition, the large disparity in gene sequences among species within the genus suggests there is a large undiscovered species diversity of *Pleuronema*.

**Fig. 1:**
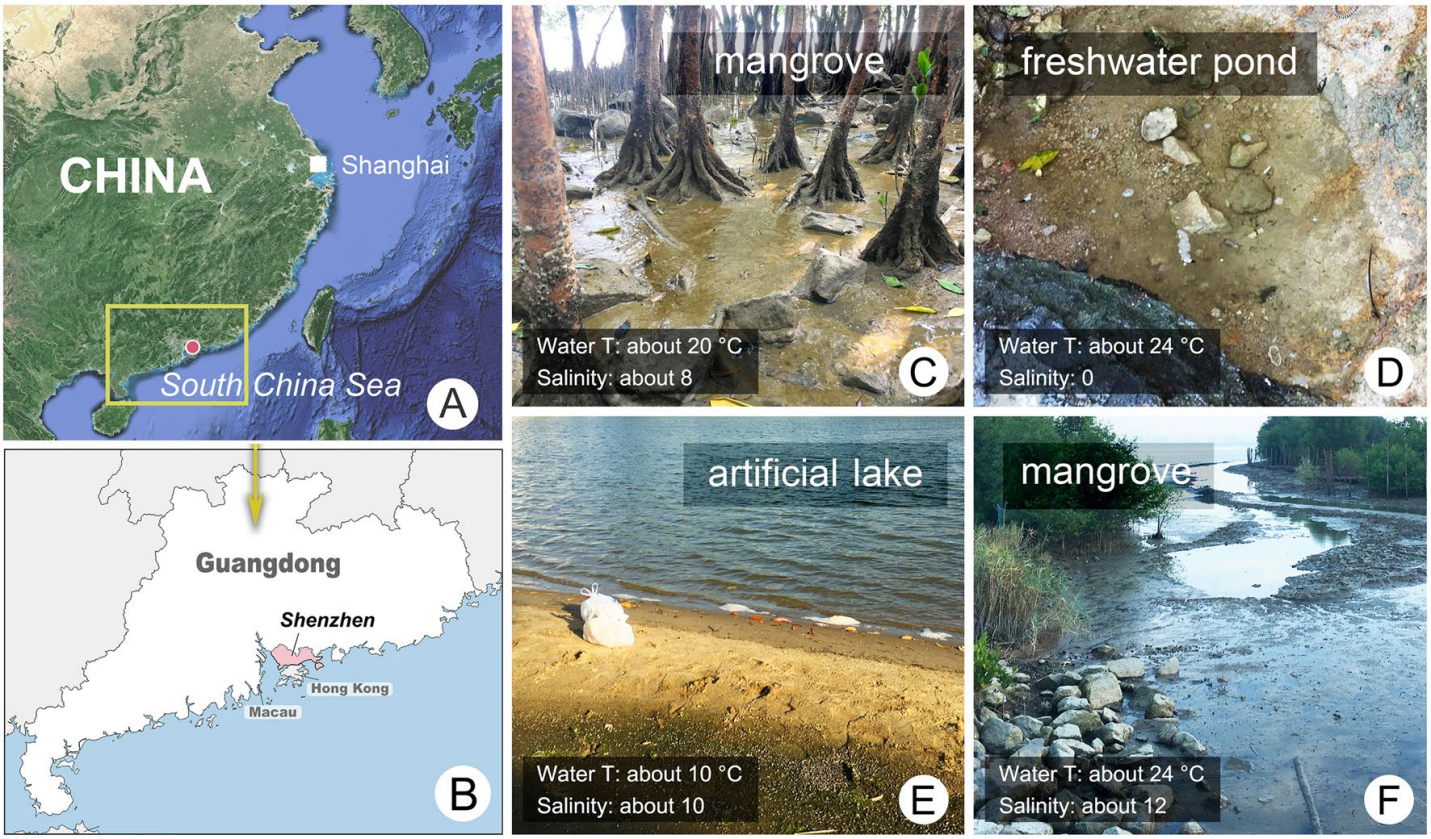
Geographic location of Shenzhen and photographs of sampling sites where the four new *Pleuronema* species were collected. **A** Portion of a map of China, red dot depicts the location of Shenzhen. **B** Map of Guangdong Province, Shenzhen is marked in pink (modified from https://d-maps.com). **C**–**F** Sampling sites and habitats of *P. foissneri* sp. nov. (**C**), *P. parasmalli* sp. nov. (**D**), *P. parasalmastra* sp. nov. (**E**) and *P. paraorientale* sp. nov. (**F**)

In the present study, four *Pleuronema* species collected from brackish and freshwater habitats in Shenzhen, southern China (Fig. 1), were investigated using modern taxonomic methods. The morphological and molecular data indicate that each of these is a new species.

## Results

### ZooBank registration

This article: urn:lsid:zoobank.org:pub:7B92C073-DD1B-4135-8121-B1818132A52E.

*Pleuronema foissneri* sp. nov.: urn:lsid:zoobank.org:act:DFB07D56-DD70-4D66-813E-BF2B669A1D13.

*Pleuronema parasmalli* sp. nov.: urn:lsid:zoobank.org:act:1D239E98-A986-4F0D-882A-8D0A6FB86650.

*Pleuronema parasalmastra* sp. nov.: urn:lsid:zoobank.org:act:75533119-0DFA-4647-85F2-37882789C453.

*Pleuronema paraorientale* sp. nov.: urn:lsid:zoobank.org:act:5F0B617D-D758-4877-B048-373ABF75D9AC.

### Taxonomy

Subclass: Scuticociliatia Small, 1967.

- Order: Pleuronematida Fauré-Fremiet in Corliss, 1956.
  - Family: Pleuronematidae Kent, 1881.
    - Genus: *Pleuronema* Dujardin, 1841.

### *Pleuronema foissneri* sp. nov. (Fig. 2; Table 1)

**Fig. 2:**
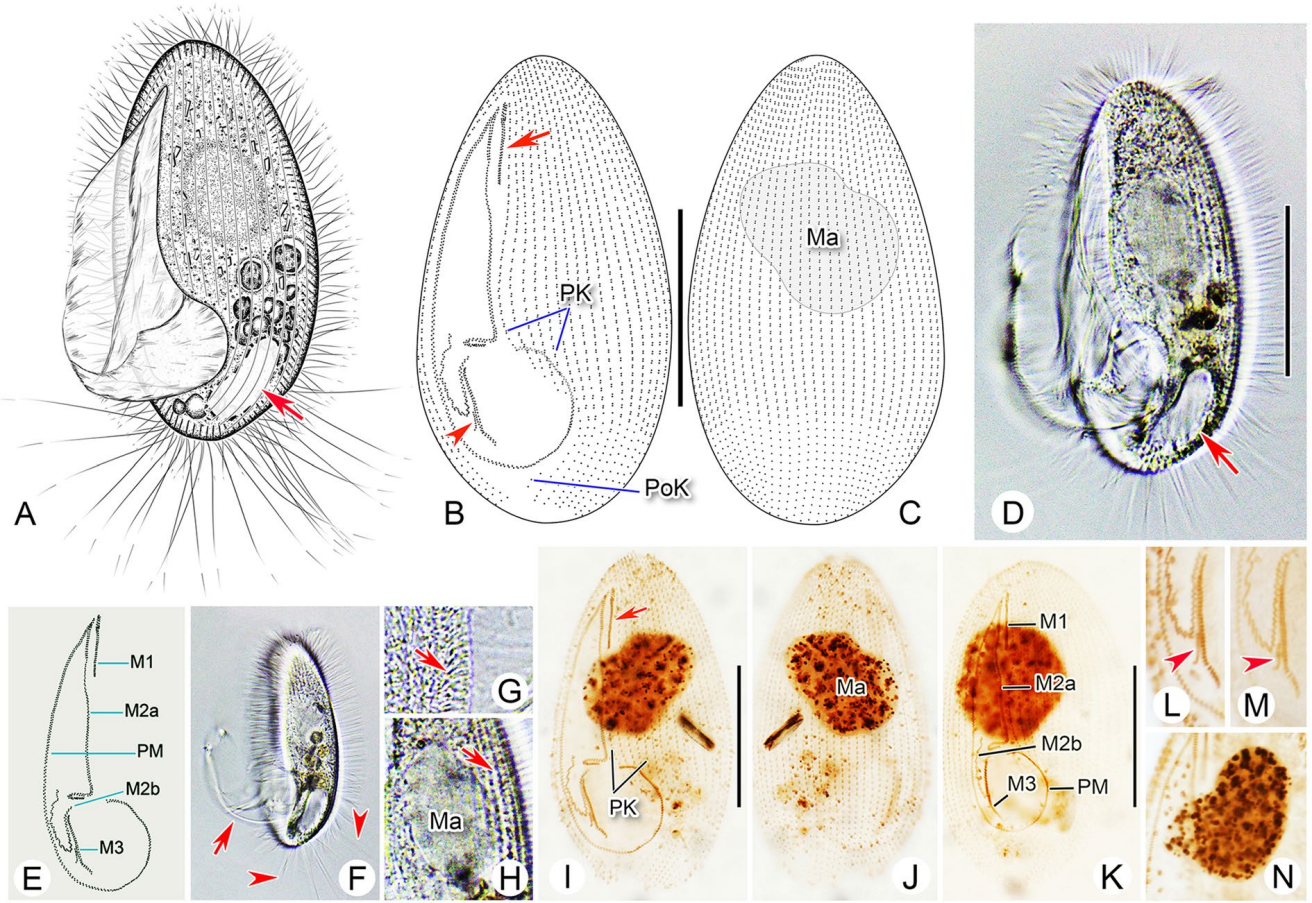
*Pleuronema foissneri* sp. nov. from life (**A**, **D**, **F**–**H**) and after protargol staining (**B**, **C**, **E**, **I**–**N**). **A** Left ventrolateral view of a representative cell, arrow shows the contractile vacuole. **B**, **C** Left ventrolateral (**B**) and right dorsolateral (**C**) views of the holotype specimen, arrow in **B** indicates membranelle 1, arrowhead in **B** depicts the shortened rightmost row of membranelle 3. **D** Left ventrolateral view of an individual in vivo, arrow points to the contractile vacuole that is not fully expanded. **E** Detail of oral structure. **F** Left lateral view of cell, arrow shows the oral cilia, arrowheads indicate the caudal cilia. **G** Detail of cortex, arrow depicts extrusomes below the pellicle. **H** Detail showing the macronucleus and the grooves on the cell surface (arrow). **I**, **J** Left ventrolateral (**I**) and right dorsolateral (**J**) views of the holotype specimen, showing the ciliature and pear-shaped macronucleus, arrow in I shows membranelle 1. **K** Ventral view of a silver-stained cell, revealing the ciliature and macronucleus. **L**, **M** Detail of oral region, arrowheads depict the shortened rightmost row of membranelle 3. **N** Showing a heart-shaped macronucleus. *M1* membranelle 1, *M2a* membranelle 2a, *M2b* membranelle 2b, *M3* membranelle 3, *Ma* macronucleus, *PK* preoral kineties, *PM* paroral membrane, *PoK* postoral kineties. Scale bars = 30 μm

**Table 1:** Morphometric data for *Pleuronema foissneri* sp. nov. (upper row) and *Pleuronema parasmalli* sp. nov. (lower row)

Characteristics	H	Min	Max	Mean	Median	SD	SE	CV	*n*
Body length (μm)	77	60	90	75.9	76	7.08	1.717	9.33	17
	74	60	80	70.4	70	4.77	0.954	6.78	25
Body width (μm)	42	35	50	41.8	42	4.72	1.145	11.29	17
	41	30	40	35.8	35	3.03	0.606	8.46	25
Oral length (μm)	60	50	68	59.3	60	4.81	1.203	8.11	16
	57	45	60	52.9	52	3.62	0.724	6.85	25
Oral length/body length	0.78	0.71	0.83	0.77	0.77	0.04	0.009	4.59	16
	0.77	0.67	0.80	0.75	0.77	0.03	0.007	4.64	25
Length of M1 (μm)	14	11	16	13.3	13	1.30	0.326	9.78	16
	10	7	11	9.4	9	0.82	0.163	8.69	25
Length of M2a (μm)	39	30	43	36.5	36	3.69	0.922	10.10	16
	35	26	38	31.6	32	3.00	0.600	9.48	25
M1 length/M2a length	0.36	0.33	0.42	0.37	0.36	0.03	0.007	7.57	16
	0.29	0.26	0.35	0.30	0.30	0.02	0.004	7.20	25
M1 length/body length	0.18	0.14	0.21	0.17	0.17	0.02	0.005	11.82	16
	0.14	0.10	0.15	0.13	0.14	0.01	0.003	9.81	25
M1 length/oral length	0.23	0.18	0.29	0.23	0.22	0.03	0.007	11.98	16
	0.18	0.13	0.20	0.18	0.18	0.02	0.004	9.93	25
M2a length/body length	0.51	0.36	0.57	0.48	0.49	0.06	0.014	11.90	16
	0.47	0.37	0.54	0.45	0.46	0.04	0.008	9.36	25
Length of M2b (μm)	14	12	17	13.9	13.5	1.50	0.375	10.81	16
	11	7	10	8.6	9	0.99	0.199	11.52	25
M2b length/body length	0.18	0.13	0.22	0.18	0.18	0.02	0.005	12.09	16
	0.15	0.09	0.15	0.12	0.12	0.01	0.003	12.15	25
Number of somatic kineties	39	32	40	36.3	36	2.40	0.621	6.63	15
	29	26	32	28.9	29	1.39	0.279	4.83	25
Number of preoral kineties	5	4	8	5.2	5	1.08	0.279	20.81	15
	6	4	6	5.2	5	0.66	0.133	12.66	25
Number of postoral kineties	1	0	1	0.3	0	0.49	0.126	146.39	15
	1	0	1	0.5	1	0.51	0.102	98.06	25
Number of kinetosome rows in M3	3	3	3	3.0	3	0	0	0	16
	3	3	3	3.0	3	0	0	0	25
Number of macronuclei	1	1	1	1.0	1	0	0	0	24
	1	1	14	1.8	1	2.67	0.534	145.22	25
Length of macronucleus (μm)	28	25	35	28.5	29	3.18	0.648	11.14	24
	16	5	22	15.4	15	3.64	0.728	23.58	25
Width of macronucleus (μm)	21	15	30	24.0	24.5	3.90	0.795	16.20	24
	–	–	–	–	–	–	–	–	–
Distance from anterior end of M1 to cell apex (μm)	7	4	10	6.9	7	1.60	0.357	23.34	20
	7	3	12	7.0	6	2.23	0.445	31.99	25
Distance from anterior end of M1 to cell apex/body length	0.09	0.06	0.13	0.09	0.09	0.02	0.004	20.94	20
	0.09	0.05	0.14	0.10	0.10	0.03	0.005	26.90	25

All data are based on randomly selected protargol-stained specimens

*CV* coefficient of variation in %, *H* holotype, *M1* membranelle 1, *M2a* membranelle 2a, *M2b* membranelle 2b, *M3* membranelle 3, *Max* maximum, *Mean* arithmetic mean, *Min* minimum, *n* number of specimens observed, *SD* standard deviation, *SE* standard error of arithmetic mean, – not applicable or data not available

*Diagnosis* Body size in vivo approximately 60–75 μm × 30–40 μm. Anterior end slightly narrowed. Right ventrolateral side convex. 32–40 somatic kineties. Four to eight preoral kineties. Macronucleus usually with a notch in mid-portion. Membranelle 1 relatively long, approximately one-sixth of cell length. Membranelle 2a in zig–zag pattern in mid-portion, posterior portion hook-like. Membranelle 3 three-rowed, rightmost row shortened. Brackish water habitat.

*Dedication* We dedicate this new species to Dr. Wilhelm Foissner (Salzburg University) in recognition of his significant contributions to the taxonomy of ciliates.

*Type locality and habitat* Brackish water from a mangrove wetland in Shenzhen Bay Park (22°31′19.8′′ N; 114°0′11.1′′ E), Shenzhen, southern China. Water temperature was approximately 20 °C and salinity was approximately 8.

*Deposition of type slides* The protargol slide containing the holotype specimen (Fig. 2B, C) and several paratype specimens (registration number: LMJ2016040401-1), and a second protargol slide containing paratype specimens (registration number: LMJ2016040401-2), were deposited in the Laboratory of Protozoology, Ocean University of China, Qingdao, China.

*Small subunit ribosomal RNA (SSU rRNA) gene sequence* The sequence of *Pleuronema foissneri* sp. nov. was deposited in GenBank with accession number OL654416. The length and G + C content of the sequence are 1637 bp and 43.01%, respectively.

*Description* Cell size in vivo approximately 60–75 μm × 30–40 μm. Elliptical or oval in outline (Fig. 2A, D, F). Anterior end slightly narrowed, posterior end rounded, right ventrolateral and dorsal sides convex (Fig. 2A, D, F). Buccal field occupying 70–85% of cell length (Fig. 2A, D; Table 1). Oral cilia approximately 30 μm long. Pellicle slightly notched with shallow longitudinal grooves (Fig. 2H). Extrusomes bar-shaped, located beneath pellicle, approximately 5 μm in length (Fig. 2E). Cytoplasm colorless to greyish, containing several food vacuoles, refractile globules and crystals that usually posteriorly distributed (Fig. 2A, D, F). Single contractile vacuole dorsally located about 85% down length of cell, approximately 8–10 μm in diameter when fully expanded, pulsating at intervals of approximately 20–40 s (Fig. 2A, D, F). Somatic cilia densely packed and approximately 8–10 μm long, perpendicular to surface when cell is at rest (Fig. 2A, D, F). Approximately 15 caudal cilia, each 25–30 μm in length. Locomotion typical of genus, i.e., usually by fast swimming with body rotating continuously about longitudinal axis.

Thirty-two to 40 somatic kineties (SK) extending almost entire length of body. Each SK consisting of dikinetids in anterior three-quarters of SK and monokinetids in posterior quarter (Fig. 2B, C, I–L; Table 1). Four to eight preoral kineties located to left of buccal field, commencing at anterior end of cell and terminating posteriorly approximately two-thirds down length of body (Fig. 2B, I; Table 1). Postoral kinety usually absent, one present in five out 15 individuals examined. Single macronucleus located one-third down length of body, approximately 25–35 μm × 15–30 μm after protargol staining, generally ellipsoidal to spherical in shape; in 14 out of 24 individuals examined, macronucleus notched in mid-portion giving it a heart- or pear-shaped appearance (Fig. 2C, I–K, N; Table 1). Micronucleus not detected.

Anterior quarter of membranelle 1 (M1) three-rowed while rest of M1 two-rowed (Fig. 2B, E, I). M1 15–20% of cell length, commencing approximately one-tenth down length of body (Fig. 2B, I, K; Table 1). Anterior one-fifth and posterior quarter of membranelle 2a (M2a) conspicuously double-rowed, basal bodies in mid-portion arranged in zig–zag pattern (Fig. 2B, E, I). Length of M2a approximately 40–60% of body length, commencing near anterior end of M1 (Fig. 2B, I, K; Table 1). Posterior portion of M2a hook-like (Fig. 2B, E, I, K). Membranelle 2b (M2b) basically V-shaped, with basal bodies arranged in several single-rowed groups in zig–zag pattern (Fig. 2B, E, I). Each group with two to six basal bodies. Length of M2b approximately 13–22% of body length, commencing at same level as posterior end of M2a (Fig. 2B, E, I, K; Table 1). Membranelle 3 composed of three densely arranged rows, rightmost row shortened, length approximately 20% of two left rows (Fig. 2B, E, I, L, M). Paroral membrane double-rowed in zig–zag pattern, occupying about 70–80% of cell length (Fig. 2B, E, I, K).

### *Pleuronema parasmalli* sp. nov. (Fig. 3; Table 1)

**Fig. 3:**
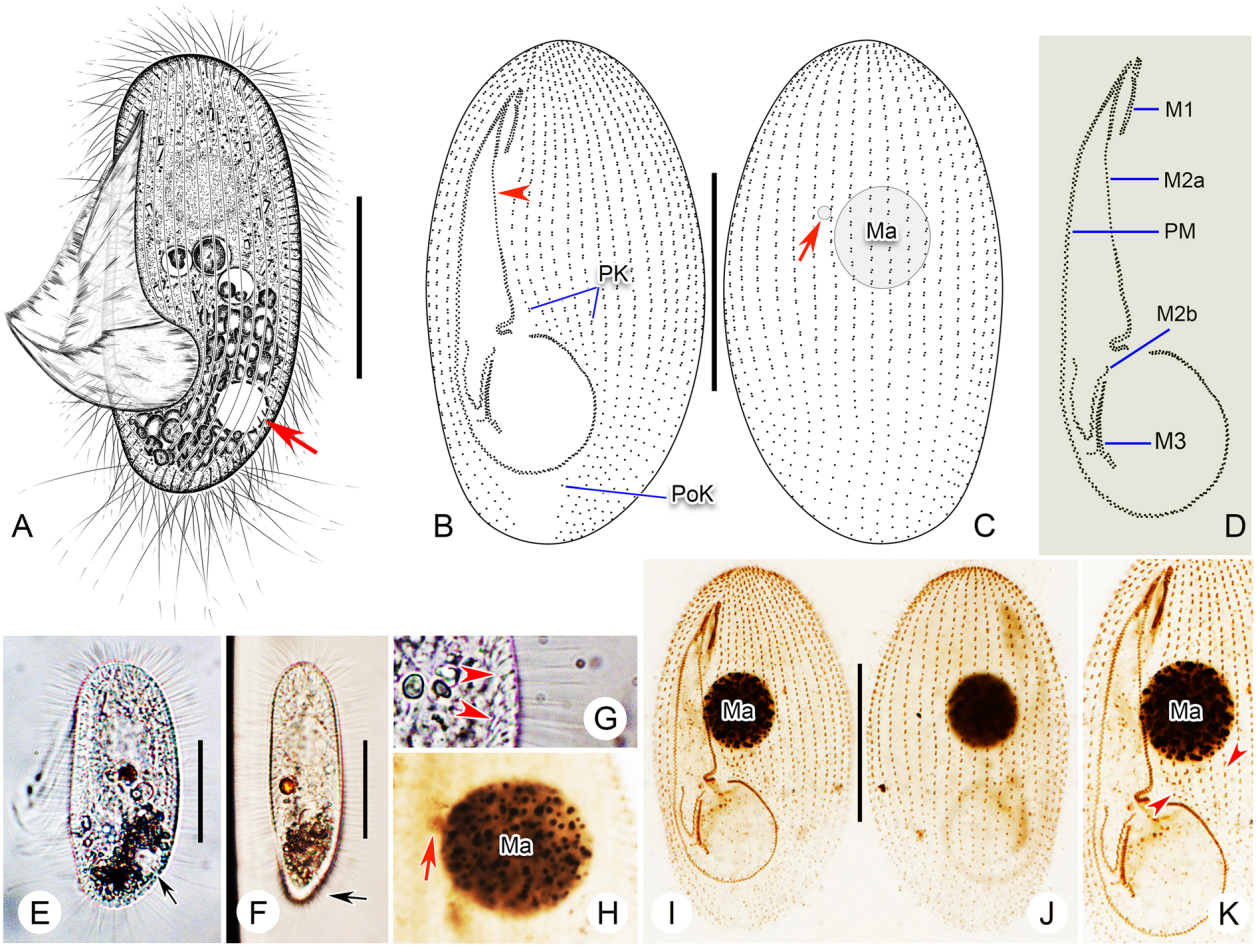
*Pleuronema parasmalli* sp. nov. from life (**A**, **E**–**G**) and after protargol staining (**B**–**D**, **H**–**K**). **A** Left ventrolateral view of a representative cell, arrow shows the contractile vacuole. **B**, **C** Left ventrolateral (**B**) and right dorsolateral (**C**) views of the holotype specimen, arrowhead in **B** indicates that the mid-portion of membranelle 2a is single-rowed, arrow in **C** points to the micronucleus. **D** Detail of oral structure. **E**, **F** Left ventrolateral (**E**) and dorsal (**F**) views of representative individuals, arrow in **E** shows the contractile vacuole, arrow in **F** indicates the narrowed posterior end. **G** Detail of cortex, arrowheads point to the extrusomes below the pellicle. **H** Nuclear apparatus, arrow shows the micronucleus. **I**, **J** Left ventrolateral (**I**) and right dorsolateral (**J**) views of the holotype specimen. **K** Ventral view, showing the oral infraciliature and macronucleus, arrowheads depict the preoral kineties. *M1* membranelle 1, *M2a* membranelle 2a, *M2b* membranelle 2b, *M3* membranelle 3, *Ma* macronucleus, *PK* preoral kineties, *PM* paroral membrane, *PoK* postoral kineties. Scale bars = 30 μm

*Diagnosis* Body 55–85 μm × 25–35 μm in vivo. Right ventrolateral side basically straight. 26–32 somatic kineties. Four to six preoral kineties. Macronucleus and micronucleus spherical. Membranelle 2a single-rowed in mid-portion, posterior portion hook-like. Freshwater habitat.

*Etymology* Composite of the Greek word *para* (beside) and the species-group name *smalli*, indicating the similarity between the new species and *Pleuronema smalli* in terms of their small body size and ciliature pattern.

*Type locality and habitat* Shallow freshwater pond at Dameisha sandy beach (22°35′12.7′′ N; 114°18′14.9′′ E) in Shenzhen, southern China. Water temperature was approximately 24 °C.

*Deposition of type slides* The protargol slide containing the holotype specimen (Fig. 3B, C) and several paratype specimens (registration number: LMJ2016111701-1), and two protargol slides containing paratype specimens (registration numbers: LMJ2016111701-2, LMJ2016111701-3), were deposited in the Laboratory of Protozoology, Ocean University of China, Qingdao, China.

*SSU rRNA gene sequence* The sequence of *Pleuronema parasmalli* sp. nov. was deposited in GenBank with accession number OL654417. The length and G + C content of the sequence are 1632 bp and 43.26%, respectively.

*Description* Body size in vivo approximately 55–85 μm × 25–35 μm. Elongate-elliptical in outline when viewed from left ventrolateral aspect (Fig. 3A, E). Anterior end rounded (Fig. 3A, E), posterior end narrowed when viewed from dorsal aspect (Fig. 3F). Laterally flattened (Fig. 3F). Right ventrolateral side basically straight, dorsal side convex (Fig. 3A, E). Length of buccal field 67–80% of body length (Fig. 3A; Table 1). Oral cilia approximately 30 μm in length. Pellicle slightly notched with shallow longitudinal grooves (Fig. 3H). Bar-shaped extrusomes densely arranged beneath pellicle and approximately 5 μm in length (Fig. 3E). Cytoplasm transparent to greyish, containing several food vacuoles, refractile globules, and crystals that are usually concentrated in posterior half of cell rendering this region black when viewed at low magnification (Fig. 3A, E, F). Single contractile vacuole dorsally located 80% down length of cell, approximately 10–12 μm across when fully expanded, pulsating at intervals of approximately 15–30 s (Fig. 3A, E). Somatic cilia densely packed, 10–12 μm in length (Fig. 3A, E–G). Approximately 15–18 caudal cilia, each 20–25 μm in length. Locomotion typical of genus, i.e., usually by fast swimming with body rotating continuously about longitudinal axis.

Twenty-six to 32 somatic kineties extending almost entire body length, each with densely spaced dikinetids in anterior three-fifths, and monokinetids in posterior two-fifths (Fig. 3B, C, I, J; Table 1). Four to six preoral kineties located to left of buccal field, commencing near anterior end of cell and terminating posteriorly 65% down length of cell (Fig. 3B, I; Table 1). Thirteen out of 25 cells examined with one postoral kinety (PoK), others without PoK. Usually (in 19 out of 25 cells examined) with a single spherical macronucleus positioned at 33 to 40% down length of body, diameter approximately 15–22 μm after protargol staining (Fig. 3C, I–K); six out of 25 cells examined with two to 14 spherical macronuclei, diameter of each varying from 5–15 μm. Single spherical micronucleus, 5–6 μm in diameter, adjacent to macronucleus (Fig. 3C, H).

First quarter of membranelle 1 (M1) three-rowed while rest two-rowed (Fig. 3B, D, I, K). Length of M1 10–15% of cell length (Fig. 3B, I; Table 1). Anterior end of M1 located approximately 10% down length of cell (Fig. 3B, I). Anterior one-eighth and posterior one-third of membranelle 2a (M2a) two-rowed, mid-portion single-rowed (Fig. 3B, D, I, K). Length of M2a 40 to 50% of body length, commencing at level of 20% down length of M1 (Fig. 3B, D, I, K). Posterior portion of M2a hook-like (Fig. 3B, D, I, K). Membranelle 2b (M2b) V-shaped, portion near each end single-rowed, basal bodies in mid-portion arranged in zig–zag pattern (Fig. 3B, D, I, K). M2b occupying approximately 10% of body length. Anterior end of M2b slightly below level of posterior end of M2a (Fig. 3B, D, I, K). Membranelle 3 with three closely packed rows, posterior end of rightmost row conspicuously separated from other rows and pointing to right (Fig. 3B, D, I, K). Paroral membrane double-rowed and in zig–zag pattern, occupying about 67–80% of cell length (Fig. 3B, D, I, K; Table 1).

### *Pleuronema parasalmastra* sp. nov. (Fig. 4; Table 2)

**Fig. 4:**
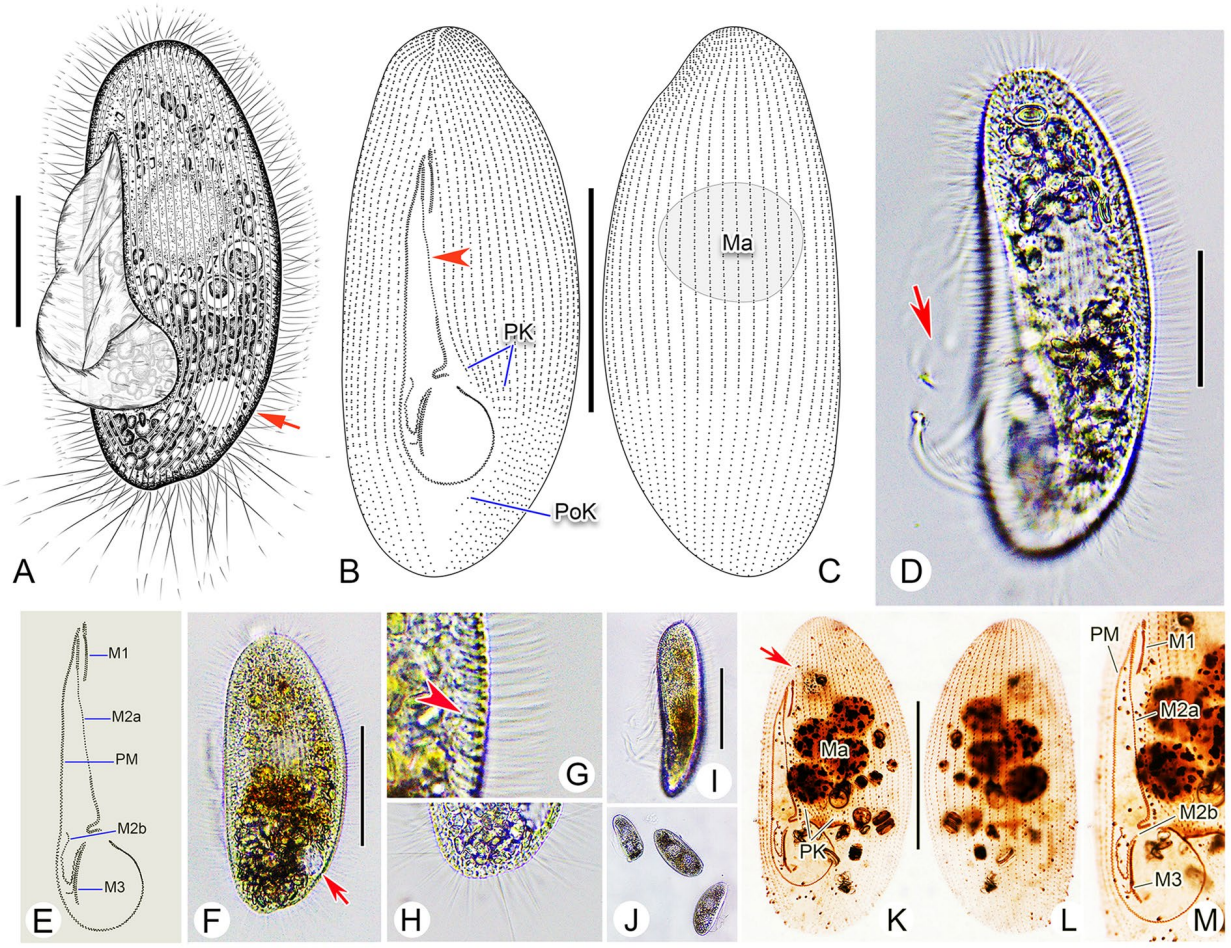
*Pleuronema parasalmastra* sp. nov. from life (**A**, **D**, **F**–**J**) and after protargol staining (**B**, **C**, **E**, **K**–**M**). **A** Left ventrolateral view of a representative cell, arrow shows the contractile vacuole. **B**, **C** Ventral (**B**) and dorsal (**C**) views of the holotype specimen, showing the ciliature and nuclear apparatus; arrowhead in **B** depicts the single-rowed mid-portion of membranelle 2a. **D** Left lateral view of a representative individual, arrow shows the oral cilia. **E** Oral ciliature. **F** Cell in vivo, arrow shows the contractile vacuole. **G** Detail of cortex, arrowhead points to the extrusomes below the pellicle. **H** Posterior end of cell, showing the caudal cilia. **I** Lateral view of cell. **J** Different individuals, showing the variation in body size. **K**, **L**, Left ventrolateral (**K**) and right dorsolateral (**L**) views of the same silver-stained specimen, arrow in **K** indicates the gap between cell apex and oral apparatus. **M** Detail of cell showing the oral ciliature. *M1* membranelle 1, *M2a* membranelle 2a, *M2b* membranelle 2b, *M3* membranelle 3, *Ma* macronucleus, *PK* preoral kineties, *PM* paroral membrane, *PoK* postoral kineties. Scale bars = 30 μm (**A**, **D**), 50 μm (**B**, **C**, **I**, **K**, **L**), 45 μm (**F**)

**Table 2:** Morphometric data for *Pleuronema parasalmastra* sp. nov. (upper row) and *Pleuronema paraorientale* sp. nov. (lower row)

Characteristics	H	Min	Max	Mean	Median	SD	SE	CV	*n*
Body length (μm)	120	95	160	119.7	118	14.13	3.648	11.80	15
	128	115	150	132.8	130	10.90	2.181	8.21	25
Body width (μm)	52	45	75	57.0	56	7.76	2.002	13.61	15
	72	60	95	76.3	75	9.75	1.951	12.79	25
Oral length (μm)	73	69	91	76.5	75	5.40	1.393	7.05	15
	89	80	100	91.0	90	6.29	1.258	6.91	25
Oral length/body length	0.61	0.57	0.74	0.64	0.64	0.04	0.009	5.66	15
	0.70	0.61	0.77	0.69	0.69	0.04	0.008	5.62	25
Length of M1 (μm)	14	12	18	14.5	14.5	1.51	0.403	10.39	14
	15	12	16	13.8	14	1.22	0.245	8.87	25
Length of M2a (μm)	47	42	58	48.8	50	4.66	1.246	9.55	14
	62	48	72	61.6	62	6.18	1.236	10.04	25
M1 length/M2a length	0.30	0.24	0.36	0.30	0.30	0.03	0.008	10.03	14
	0.24	0.19	0.25	0.23	0.22	0.02	0.004	8.54	25
M1 length/body length	0.12	0.10	0.13	0.12	0.12	0.01	0.002	6.78	14
	0.12	0.08	0.13	0.10	0.10	0.01	0.002	10.43	25
M1 length/oral length	0.19	0.16	0.21	0.19	0.19	0.01	0.004	6.95	14
	0.17	0.13	0.18	0.15	0.15	0.01	0.002	7.68	25
M2a length/body length	0.39	0.34	0.45	0.41	0.42	0.03	0.008	6.96	14
	0.48	0.37	0.58	0.47	0.46	0.06	0.011	12.28	25
Length of M2b (μm)	14	12	18	13.3	13	1.64	0.438	12.33	14
	26	20	32	26.0	26	3.38	0.677	13.00	25
M2b length/body length	0.12	0.10	0.13	0.11	0.11	0.01	0.002	5.87	14
	0.20	0.15	0.28	0.20	0.19	0.03	0.006	15.50	25
Number of somatic kineties	37	37	43	39.8	39	1.57	0.405	3.94	15
	53	52	62	56.1	56	2.85	0.570	5.07	25
Number of preoral kineties	6	4	6	5.6	6	0.63	0.163	11.29	15
	4	3	5	3.7	4	0.74	0.147	19.81	25
Number of postoral kineties	1	0	1	0.7	1	0.48	0.153	69.01	10
	2	0	3	1.2	1	0.54	0.107	47.10	26
Number of kinetosome rows in M3	3	3	3	3.0	3	0	0	0	13
	3	3	3	3.0	3	0	0	0	25
Number of macronuclei	1	1	6	1.8	1	1.48	0.371	84.76	16
	1	1	12	1.5	1	2.22	0.444	146.03	25
Length of macronucleus (μm)	33	15	36	25.8	26.5	6.42	1.606	24.95	16
	39	15	45	34.7	35	7.31	1.461	21.07	25
Number of micronuclei	–	–	–	–	–	–	–	–	–
	3	2	10	4.3	3.5	1.95	0.357	45.08	30
Diameter of micronucleus (μm)	–	–	–	–	–	–	–	–	–
	4	3	6	4.5	4	1.04	0.312	23.25	11
Distance from anterior end of M1 to cell apex (μm)	26	16	35	26.0	25	4.62	0.925	17.81	25
	16	8	24	16.0	16	3.51	0.701	21.86	25
Distance from anterior end of M1 to cell apex/body length	0.22	0.16	0.25	0.21	0.21	0.02	0.005	11.17	25
	0.13	0.07	0.16	0.12	0.12	0.02	0.004	17.75	25

All data are based on randomly selected protargol-stained specimens

*CV* coefficient of variation in %, *H* holotype, *M1* membranelle 1, *M2a* membranelle 2a, *M2b* membranelle 2b, *M3* membranelle 3, *Max* maximum, *Mean* arithmetic mean, *Min* minimum, *n* number of specimens observed, *SD* standard deviation, *SE* standard error of arithmetic mean, – not applicable or data not available

*Diagnosis* Body size in vivo approximately 90–120 μm × 40–55 μm. Right ventrolateral side straight. 37–43 somatic kineties. Four to six preoral kineties. Membranelle 1 commences about 20% down length of cell. Membranelle 2a single-rowed in mid-portion, posterior portion hook-like. Brackish water habitat.

*Etymology* Composite of the Greek word *para* (beside) and the species-group name *salmastra*, indicating that the new species resembles the large individuals of *Pleuronema salmastra* in having an entirely posterior-positioned buccal field.

*Type locality and habitat* Brackish water from Yelin Sand Beach (22°31′19.5′′ N; 113°59′17.2" E), Shenzhen, southern China. Water temperature was approximately 10 °C and salinity was approximately 10.

*Deposition of type slides* The protargol slide containing the holotype specimen (Fig. 4B, C) and several paratype specimens (registration number: LMJ2015121701-1), and two protargol slides containing paratype specimens (registration numbers: LMJ2015121701-2, LMJ2015121701-3), were deposited in the Laboratory of Protozoology, Ocean University of China, Qingdao, China.

*SSU rRNA gene sequence* The sequence of *Pleuronema parasalmastra* sp. nov. was deposited in GenBank with accession number OL654418. The length and G + C content of the sequence are 1630 bp and 44.36%, respectively.

*Description* Body size in vivo approximately 90–120 μm × 40–55 μm. Elongate-elliptical in outline (Fig. 4A, D, F). Anterior and posterior ends rounded, right ventrolateral side straight, dorsal side convex (Fig. 4A, D, F). Length of buccal field approximately 60–75% of body length (Fig. 4A, D; Table 2). Oral cilia approximately 25 μm long. Pellicle slightly notched with shallow longitudinal grooves. Extrusomes bar-shaped and densely distributed beneath pellicle, approximately 4–5 μm long (Fig. 4G). Cytoplasm greyish to yellowish-brown, containing numerous food particles including algae, usually distributed in anterior and posterior portions of cell (Fig. 4A, D, F). Single contractile vacuole dorsally located 80% down length of cell, approximately 15 μm across when fully expanded, pulsating at intervals of approximately 60–120 s (Fig. 4A, F). Somatic cilia densely packed, radiating from cell surface, approximately 10 μm in length (Fig. 4A, D, G). Approximately 18–20 caudal cilia, each 25–30 μm in length. Locomotion typical of genus, i.e., usually by fast swimming with body rotating continuously about longitudinal axis.

Thirty-seven to 43 somatic kineties extending entire body length, each with closely arranged dikinetids in anterior 33–75% portion, and monokinetids in remaining portion (Fig. 4B, C, K, L; Table 2). Four to six preoral kineties located to left of buccal field, commencing near anterior end of cell and terminating posteriorly 75% down length of cell (Fig. 4B, K; Table 2). One postoral kinety in seven out of ten cells examined, postoral kinety lacking in remaining three cells (Fig. 4B). Usually (in 12 out of 16 cells examined) with a single ellipsoidal macronucleus, centrally positioned, approximately 23–36 μm in length after protargol staining (Fig. 4C); four out of 16 cells examined with three to six spherical macronuclei, each approximately 15–20 μm across (Fig. 4K, L). Micronucleus not detected.

Anterior 20% of membranelle 1 (M1) three-rowed, posterior 80% two-rowed (Fig. 4B, E, M). Length of M1 10–13% of cell length (Fig. 4K; Table 2). M1 commencing approximately 15 to 25% down length of cell (Fig. 4B, K). Anterior 20% and posterior 25% of membranelle 2a (M2a) two-rowed, remaining portion single-rowed (Fig. 4B, E, M). Length of M2a occupying approximately 33 to nearly 50% of body length, commencing slightly lower than anterior end of M1 (Fig. 4B, E, M). Posterior portion of M2a hook-like (Fig. 4B, E, K, M). Membranelle 2b (M2b) V-shaped, first quarter on right side single-rowed, basal bodies in remaining portion arranged in zig–zag pattern (Fig. 4B, E, M). M2b occupying approximately 10% of body length (Fig. 4B, K). Anterior end of M2b and posterior end of M2a at approximately same level (Fig. 4B, E, M). Membranelle 3 three-rowed and closely packed (Fig. 4B, E, M). Paroral membrane double-rowed in zig–zag pattern, occupying about 60–75% of cell length (Fig. 4B, E, K).

### *Pleuronema paraorientale* sp. nov. (Fig. 5; Table 2)

**Fig. 5:**
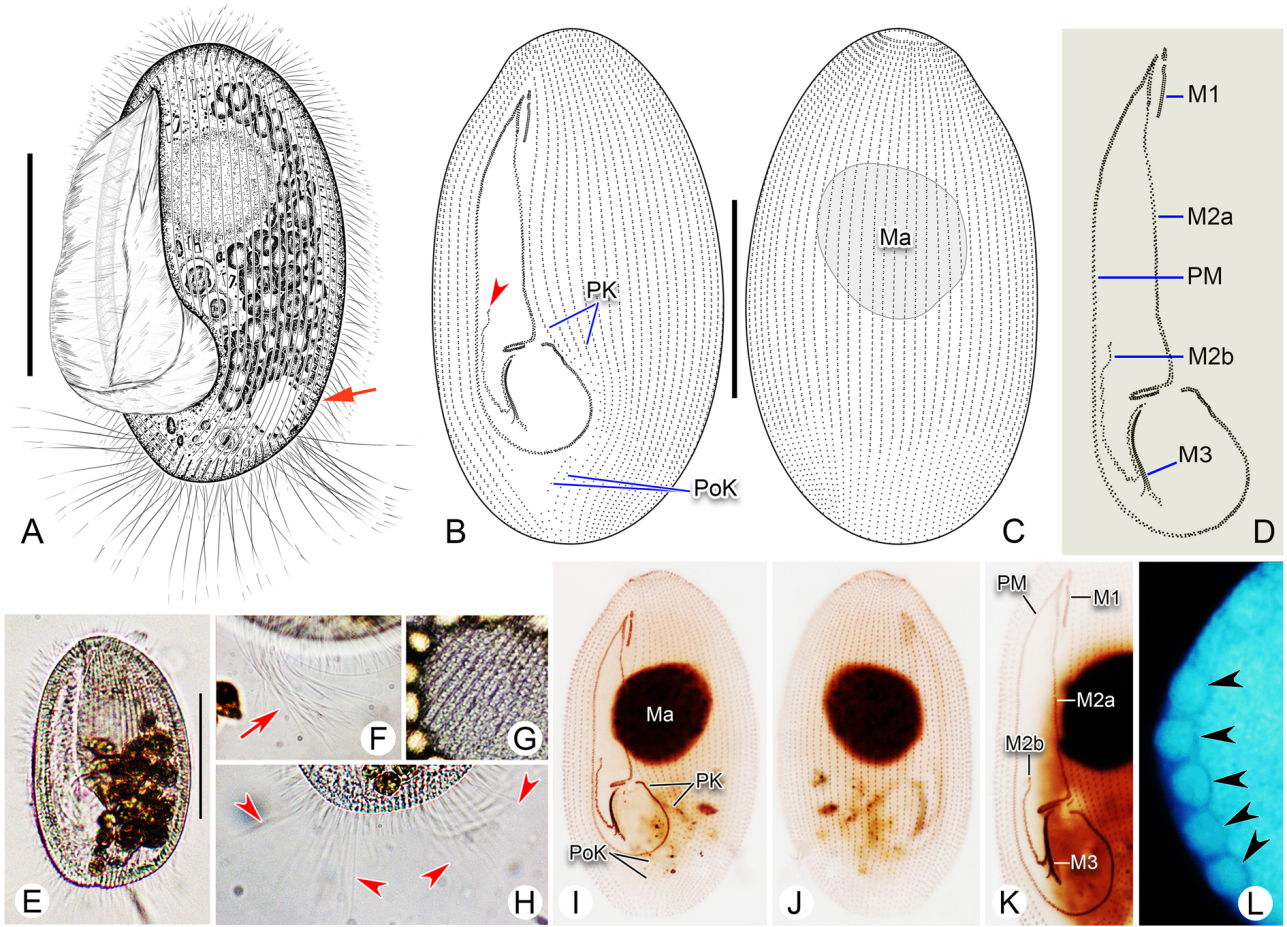
*Pleuronema paraorientale* sp. nov. from life (**A**, **E**–**H**) and after protargol staining (**B**–**D**, **I**–**L**). **A** Left ventrolateral view of a representative cell, arrow shows the contractile vacuole. **B**, **C** Left ventrolateral (**B**) and right dorsolateral (**C**) views of the holotype specimen, showing the ciliature and nuclear apparatus; arrowhead in **B** indicating that membranelle 2b commences significantly above the level of the posterior end of membranelle 2a. **D** Details of oral ciliature. **E** Left lateral view of an individual in vivo. **F**, **H** Caudal cilia on ventral (**F**, arrow) and dorsal (**H**, arrowheads) side of cell. **G** Detail of pellicle, showing the grooves along the ciliary rows. **I**, **J** Left ventrolateral (**I**) and right dorsolateral (**J**) views of the holotype specimen. **K** Detail of cell showing the oral ciliature. **L** Details of nuclear apparatus (color-inverted by Adobe Photoshop), arrowheads point to the multiple micronuclei adjacent to macronucleus. *M1* membranelle 1, *M2a* membranelle 2a, *M2b* membranelle 2b, *M3* membranelle 3, *Ma* macronucleus, *PK* preoral kineties, *PM* paroral membrane, *PoK* postoral kineties. Scale bars = 50 μm

*Diagnosis* Body size in vivo approximately 95–115 μm × 55–70 μm. Right ventrolateral side convex. 52–62 somatic kineties. Three to five preoral kineties. Two to ten spherical micronuclei. Membranelle 2a hook-like in posterior portion, basal bodies in mid-portion arranged in zig–zag pattern. Membranelle 2b commences significantly above level of posterior end of membranelle 2a. Brackish water habitat.

*Etymology* Composite of the Greek word *para* (beside) and the species-group name *orientale*, indicating that this species resembles *Pleuronema orientale* in body size, shape and ciliature pattern.

*Type locality and habitat* Brackish water from a mangrove wetland on the west coast of Shenzhen Bay (22°30′8.2′′ N; 113°57′10.7′′ E), Shenzhen, southern China. Water temperature was approximately 24 °C, salinity was approximately 12.

*Deposition of type slides* The protargol slide containing the holotype specimen (Fig. 5B, C) and several paratype specimens (registration number: LMJ2017010601-1), and one protargol slide containing paratype specimens (registration number: LMJ2017010601-2), were deposited in the Laboratory of Protozoology, Ocean University of China, Qingdao, China.

*SSU rRNA gene sequence* The sequence of *Pleuronema paraorientale* sp. nov. was deposited in GenBank with accession number OL654419. The length and G + C content of the sequence are 1639 bp and 43.50%, respectively.

*Description* Body size in vivo approximately 95–115 μm × 55–70 μm. Broad-elliptical in lateral view (Fig. 5A, E). Anterior and posterior ends rounded (Fig. 5A, E). Right ventrolateral and dorsal sides convex (Fig. 5A, E). Length of buccal field approximately 60–80% of body length (Fig. 5A; Table 2). Oral cilia approximately 40 μm in length. Pellicle slightly notched with shallow longitudinal grooves (Fig. 5G). Extrusomes bar-shaped, approximately 5 μm in length, densely distributed beneath pellicle. Cytoplasm grey to yellowish, making cells appear brown in color at low magnification (Fig. 5E). Several food vacuoles, refractile globules and crystals usually concentrated in posterior part of cell (Fig. 5E). Single contractile vacuole dorsally located approximately 80% down length of cell, diameter 10–12 μm when fully expanded, pulsating at intervals of 40–60 s (Fig. 5A, E). Somatic cilia densely arranged, radiating from cell surface, 10–12 μm long (Fig. 5A, E). Twenty to 30 caudal cilia, each 30–40 μm in length (Fig. 5F, H). Locomotion by fast swimming with body rotating continuously about longitudinal axis.

Fifty-two to 62 somatic kineties (SK) extending almost entire body length, with slender glabrous area at anterior end of cell (Fig. 5B, C, I, J; Table 2). Each SK with close-set dikinetids in anterior 80%, and monokinetids in posterior 20% (Fig. 5B, C, I, J). Three to five preoral kineties located to left of buccal field, commencing near anterior end of cell and terminating posteriorly approximately 65% down length of cell (Fig. 5B, I; Table 2). Zero to three postoral kineties (PoK) located near right side of posterior end of SKn (first kinety on left of buccal field): among 26 examined cells, one cell without PoK, 21 cells with one PoK, three cells with two PoK, and one cell with three PoK, (Fig. 5B, I; Table 2). Usually (in 23 out of 25 cells examined) with a single ellipsoidal macronucleus positioned 40% down length of cell, approximately 25–45 μm in length after protargol staining (Fig. 5C, I, J); occasionally (in two out of 25 cells examined) with three or 12 spherical macronuclei, each approximately 15–20 μm in diameter. Two to ten (usually four) spherical micronuclei, each approximately 3–6 μm across, usually adjacent to macronucleus (Fig. 5L; Table 2).

Membranelle 1 (M1) three-rowed in anterior 15 to 20% portion, two-rowed in remaining portion (Fig. 5B, D, I, K). Length of M1 8–13% of cell length (Fig. 5B, I; Table 2). M1 commencing about 12% down body length (Fig. 5B, I). Anterior 15% and posterior 33% of membranelle 2a (M2a) two-rowed, basal bodies in remaining portion arranged in a zig–zag pattern (Fig. 5B, D, I, K). M2a occupying approximately 40–60% of body length, commencing near level of anterior end of M1 (Fig. 5B, I; Table 2). Posterior portion of M2a hook-like, located approximately 60% down length of cell (Fig. 5B, D, I, K). Membranelle 2b (M2b) V-shaped, basal bodies in right anterior 20% arranged in a continuous single row, those in remaining portion arranged in several single-rowed groups, each group composed of two to six basal bodies and linked with other groups (Fig. 5B, D, I, K). M2b occupying approximately 15 to 28% of body length, commencing significantly above level of posterior end of M2a (Fig. 5B, D, I, K). Membranelle 3 three-rowed and closely packed, posterior end of rightmost row slightly separated from other rows by diverging rightwards (Fig. 5B, D, I, K). Paroral membrane (PM) double-rowed, basal bodies arranged in zig–zag pattern, occupying approximately 60–80% of cell length (Fig. 5B, D, I, K).

### Molecular data and phylogenetic analyses (Figs. 6, 7; Supplementary Table S1)

**Fig. 6:**
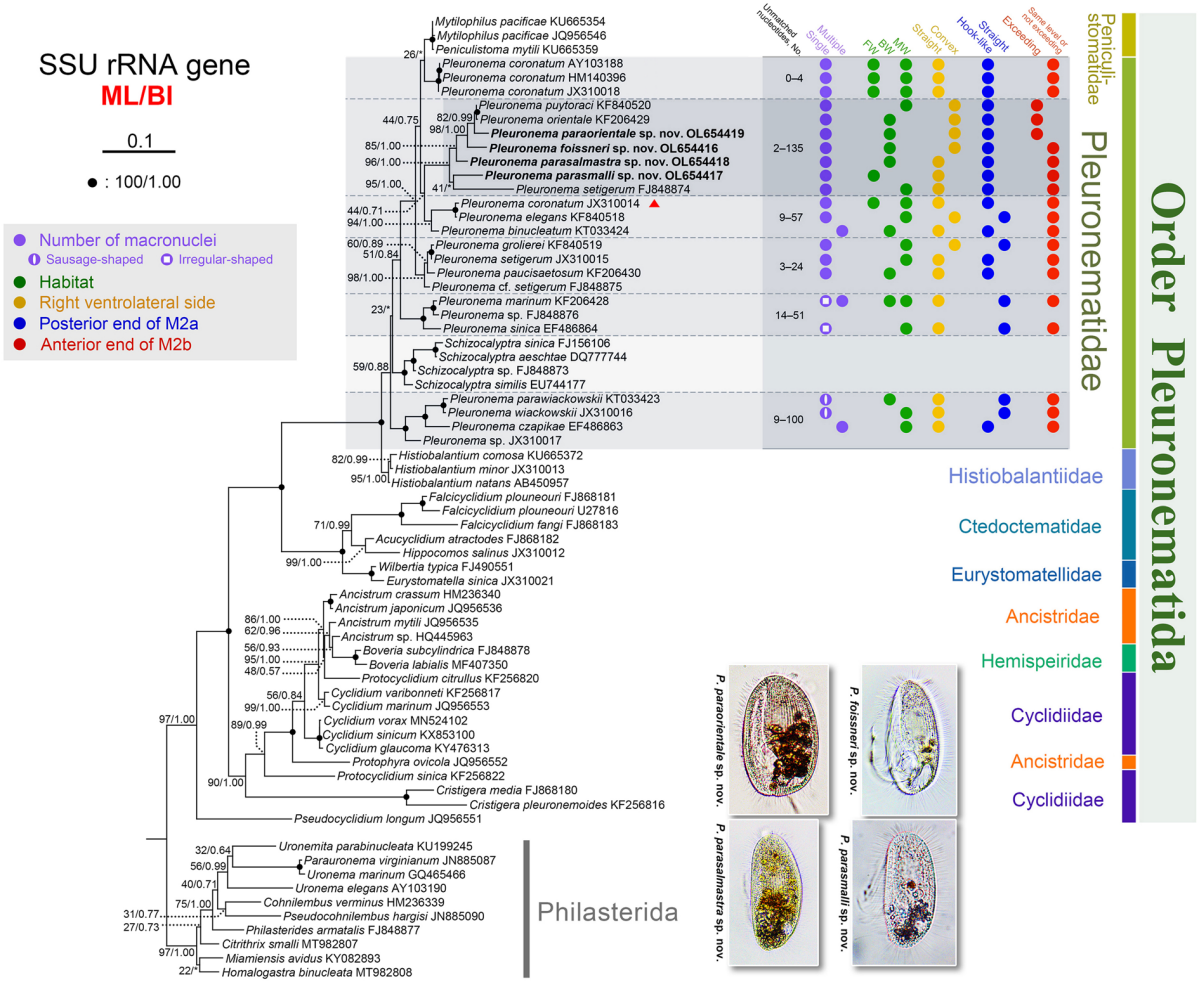
Maximum likelihood (ML) tree based on SSU rRNA gene sequence data, showing the phylogenetic positions of the four new *Pleuronema* species (in bold). Numbers at nodes denote ML bootstrap values/Bayesian inference (BI) posterior probabilities. Asterisks (*) indicate mismatch in topologies between ML and BI analyses. Fully supported (100%/1.00) nodes are marked with solid circles. The scale bar corresponds to 10 substitutions per 100 nucleotide positions. All branches are drawn to scale. The systematic classification mainly follows Gao et al. (2016). *Pleuronema coronatum* JX310014 (marked with red triangle) deviates from the other three *P. coronatum* sequences, unfortunately, available information is not sufficient to determine its identity. Main morphological features of *Pleuronema* species and the number of unmatched nucleotides within each *Pleuronema* clade are both provided. “Exceeding” in anterior end of M2b means that M2b commences above level of posterior end of M2a, while “not exceeding” means it commences below level of posterior end of M2a. *BW* brackish water, *FW* freshwater, *M2a* membranelle 2a, *M2b* membranelle 2b, *MW* marine water

**Fig. 7:**
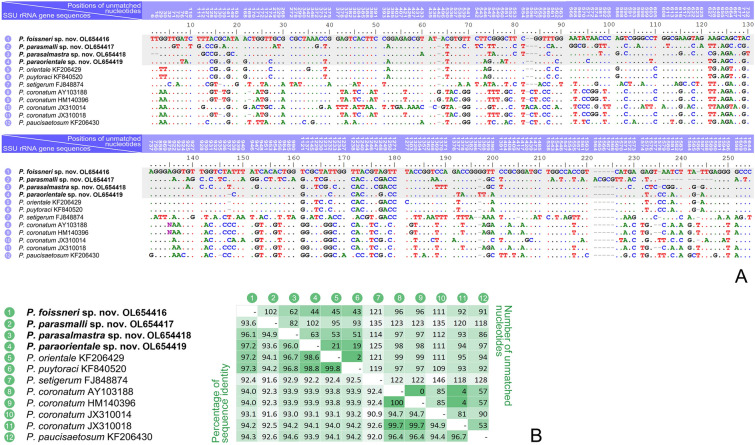
Comparison of SSU rRNA gene sequences of four new species with their most closely related congeners. **A** SSU rRNA gene comparison, showing the positions (given at the top of each column) of unmatched columns in the alignment. Insertions and deletions are compensated by introducing alignment gaps (–). **B** Matrix showing the percentage of sequence identity (below the diagonal) and the number of unmatched nucleotides (above the diagonal)

The topologies of the maximum likelihood (ML) and Bayesian inference (BI) trees were similar, therefore, only the ML tree, with support values from both algorithms, is shown (Fig. 6). The order Pleuronematida is monophyletic with high support (97% ML/1.00 BI). Within the Pleuronematida, the families Ctedoctematidae and Eurystomatidae form a fully supported clade that branches first, followed by the family Histiobalantiidae. The family Peniculistomatidae is nested within the family Pleuronematidae forming a single clade (59% ML/0.88 BI). Within this clade, members of the genera *Schizocalyptra*, *Mytilophilus*, and *Peniculistoma* are scattered among members of *Pleuronema*, making the genus *Pleuronema* polyphyletic.

All four newly sequenced *Pleuronema* species group with *P. puytoraci* KF840520, *P. orientale* KF206429 and *P. setigerum* FJ848874 with high support (96% ML/1.00 BI) (Fig. 6). Among these, *P. paraorientale* sp. nov. OL654419 clusters with *P. orientale* KF206429 and *P. puytoraci* KF840520 (87% ML/1.00 BI), followed by *P. foissneri* sp. nov. OL654416 and *P. parasalmastra* sp. nov. OL654418. In the ML tree, *P. parasmalli* sp. nov. OL654417 clusters with *P. setigerum* FJ848874 but with very low support (41% ML), forming a sister clade to the *P. puytoraci* + *P. parasalmastra* clade, whereas in the BI tree, *P. parasmalli* sp. nov. OL654417 is sister to the *P. puytoraci* + *P. parasalmastra* clade with low support (0.54 BI).

A comparison of all SSU rRNA gene sequences of *Pleuronema* species (Supplementary Table S1) shows that the intraspecific sequence identities are 94.7–100% with 0–85 unmatched nucleotides (for four *P. coronatum* sequences) and 91.6% with 135 unmatched nucleotides (two *P. setigerum* sequences). In contrast, the interspecific sequence identities range from 88.8 to 99.8%, with two (*P. puytoraci* KF840520 and *P. orientale* KF206429) to 180 (*P. setigerum* FJ848874 and *P. parawiackowskii* KT033423) unmatched nucleotides (Supplementary Table S1).

The SSU rRNA gene sequence *Pleuronema foissneri* sp. nov. OL654416 largely resembles those of *P. puytoraci* KF840520, *P. paraorientale* sp. nov. OL654419, and *P. orientale* KF206429, with 43–45 unmatched nucleotides and sequence identities of 97.2–97.3% (vs. 62–151 unmatched nucleotides and 90.6–96.1% sequence identities when compared with other *Pleuronema* sequences) (Fig. 7; Supplementary Table S1).

*Pleuronema parasmalli* sp. nov. OL654417 is most similar to *P. parasalmastra* sp. nov. OL654418, although the number of unmatched nucleotides is 82 (sequence identity 94.9%) (Fig. 7; Supplementary Table S1). In addition, *P. parasmalli* sp. nov. OL654417 seems to be more divergent from other sequences of the *P. puytoraci* + *P. setigerum* FJ848874 clade (with 82–135 unmatched nucleotides). In contrast, the other three new sequences (OL654416, OL654418, OL654419) differ from that clade by 19–125 unmatched nucleotides (Fig. 7).

The SSU rRNA gene sequence *Pleuronema parasalmastra* sp. nov. OL654418 is most similar to *P. puytoraci* KF840520 and *P. orientale* KF206429, with 51 and 53 unmatched nucleotides (96.8% and 96.7% sequence identity), respectively. It differs from other *Pleuronema* sequences in 62–151 nucleotides (90.6–96.1% sequence identity) (Fig. 7; Supplementary Table S1).

*Pleuronema paraorientale* sp. nov. OL654419 is most similar to *P. puytoraci* KF840520 and *P. orientale* KF206429, with 19 and 21 unmatched nucleotides (98.8% and 98.6% sequence identity), respectively, while there are 44–154 different nucleotides (90.4–97.2% sequence identity) compared with sequences of other congeners (Fig. 7; Supplementary Table S1).

## Discussion

### Comments on *Pleuronema coronatum* Kent, 1881

*Pleuronema coronatum* is one of the most common and well-studied species of *Pleuronema*. Since its establishment based on a freshwater population (Kent 1881), several populations isolated from freshwater, brackish water or marine water, mainly collected from Europe, North America, Africa, and East Asia, have been reported under the name “*P. coronatum*” (Agamaliev 1968; Borror 1963, 1972; Chorik 1968; Dragesco 1960, 1968; Dragesco and Dragesco-Kernéis 1986; Foissner et al. 1994; Kahl 1926, 1931; Noland 1937; Small and Lynn 1985; Song 2000; Wang et al. 2008a). This species, which was originally called “*Pleuronema coronata”*, is shorter and thicker than the morphologically related congener “*Pleuronema chrysalis*”, and has extrusomes and caudal cilia, both of which are absent in the latter (Ehrenberg 1838; Kent 1881). In addition, the former has a straight right ventrolateral side and a spherical macronucleus according to the original drawing of a cell in vivo (Kent 1881), which we consider to be diagnostic features of *P. coronatum*.

The body length in vivo of two populations of *P. coronatum* described by Wang et al. (2008a) range from 55 to 170 μm, the extremes of which deviate significantly from the original description (approximately 90 μm in length), suggesting that these populations may have included multiple species. It is noteworthy that Wang et al. (2008a) regarded *Pleuronema balli*, *P. borrori*, and *P. smalli* as synonyms of *P. coronatum* since they share some similar characteristics. After reinvestigating those descriptions, we agree that *P. balli* is a synonym of *P. coronatum*, since the former matches well with the original and other reliable populations of the latter in terms of body size, ciliature pattern, and macronucleus shape (Borror 1963; Chorik 1968; Dragesco 1968; Foissner et al. 1994; Kent 1881; Small and Lynn 1985; Song 2000). As a result, both *P. balli* populations (Dragesco 1968; Small and Lynn 1985) should be considered as *P. coronatum* populations. However, we disagree that *Pleuronema borrori* and *P. smalli* are synonyms of *P. coronatum* (Wang et al. 2008a). *Pleuronema borrori* has a much wider body (70–77 μm after silver staining vs. usually 25–65 μm in *P. coronatum*) and a smaller ratio of buccal field/body length (approximately 0.55 in *P. borrori* vs. 0.64–0.75, data measured from the drawings of silver-stained cells) than *P. coronatum* (Borror 1963; Dragesco 1968; Foissner et al. 1994; Small and Lynn 1985; Song 2000). *Pleuronema smalli* has fewer somatic kineties than *P. coronatum* (28–36 vs. 35–48) and an ellipsoid macronucleus (vs. spherical in *P. coronatum*) (Borror 1963, 1972; Chorik 1968; Dragesco 1968; Foissner et al. 1994; Kent 1881; Small and Lynn 1985; Song 2000). Hence, we recognize *P. borrori* and *P. smalli* as valid species.

In summary, *Pleuronema coronatum* can be characterized as follows: body size in vivo usually 60–125 μm × 30–60 μm; right ventrolateral side straight; 35–48 somatic kineties; three to seven preoral kineties; single spherical macronucleus; one to three micronuclei; membranelle 2a usually arranged in a zig–zag pattern in mid-portion, posterior region hook-like; freshwater and marine habitat (Borror 1963; Chorik 1968; Dragesco 1968; Foissner et al. 1994; Kent 1881; Small and Lynn 1985; Song 2000).

### Comments on *Pleuronema foissneri* sp. nov. (Fig. 8; Table 3)

**Fig. 8:**
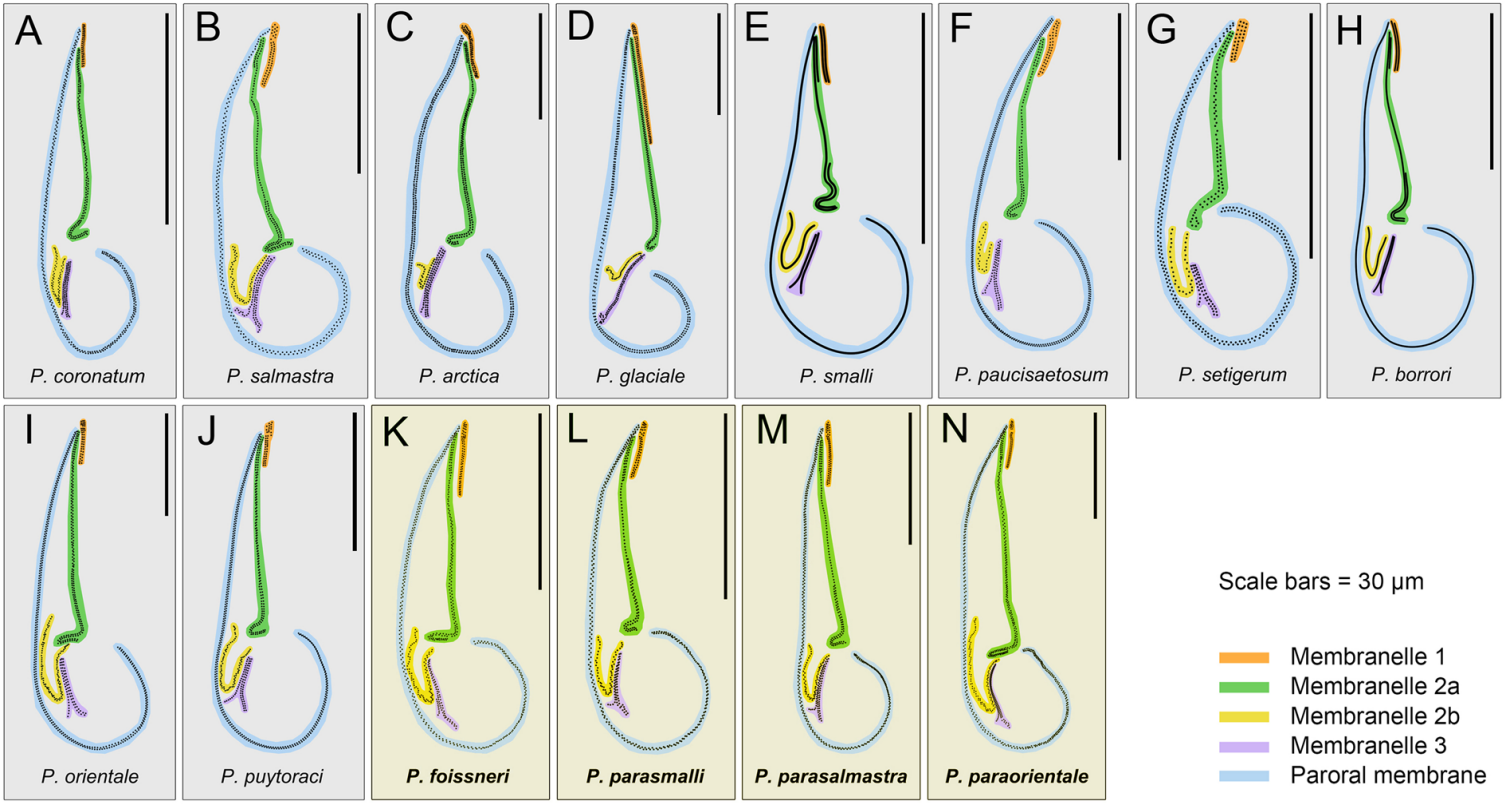
Comparison of the oral ciliature of the four new species (yellow blocks) with the related *Pleuronema* species (grey blocks, redrawn from previous studies). **A** *P. coronatum* (from Song 2000). **B** *P. salmastra* (from Dragesco and Dragesco-Kernéis 1986). **C** *P. arctica* (from Agatha et al. 1993). **D** *P. glaciale* (from Corliss and Snyder 1986). **E** *P. smalli* (from Dragesco 1968). **F** *P. paucisaetosum* (from Pan et al. 2015a). **G** *P. setigerum* (from Pan et al. 2010). **H** *P. borrori* (from Dragesco 1968). **I** *P. orientale* (from Pan et al. 2015a). **J** *P. puytoraci* (from Groliere and Detcheva 1974). **K**–**N** The four new species described in the present work. Scale bars = 30 μm (scale bar in J is inferred from the average length of membranelle 1 in Groliere and Detcheva 1974)

**Table 3:** Morphological comparison of *Pleuronema foissneri* sp. nov. with related congeners

Characteristics	*P. foissneri*	*P. coronatum*	*P. salmastra*	*P. glaciale*	*P. arctica*
Data source	Present work	[1–7]	[8]	[9]	[10]
Body size in vivo (μm)	60–75 × 30–40	60–125 × 30–60	–	–	–
Body size after staining (μm)	60–90 × 35–50	50–115 × 25–65	50–116 × 22–40	125–168 × 42–60	131–242 × 48–116
Right ventrolateral side in vivo	Convex	Straight	Straight	–	–
Length of caudal cilia	25–30 μm	About 30 μm	–	–	Up to 21 μm
Number of caudal cilia	About 15	10–15	10^a^	–	–
Extrusomes	Present	Present	Present	–	Present
Number of SK	32–40	35–48	43–63	44–58	39–61
Number of PK	4–8	3–7	6–10	6	3–4
Length ratio of M1/M2a	0.33–0.42	0.16^a^–0.36^a^	0.19^a^–0.28^a^	About 0.50 (0.58^a^)	0.26^a^
Length ratio of M1/body	0.14–0.21 (avg. 0.17)	0.08^a^–0.17^a^	0.09^a^–0.10^a^	0.33^a^	0.13^a^
Shape of Ma	Spherical to ellipsoidal, usually with a notch in mid-portion	Spherical	Spherical to ellipsoidal	–	–
Number of Mi	–	1–3	1–14, avg. 4.8 (*n* = 114)	1	1
Habitat	Brackish water (salinity 8)	Freshwater and marine water	Brackish water	Marine water	Marine water

*avg.* average, *M1* membranelle 1, *M2a* membranelle 2a, *Ma* macronucleus, *Mi* micronuclei, *n* number of examined cells, *PK* preoral kineties, *SK* somatic kineties, – information not available. References: [1] Borror (1963), [2] Chorik (1968), [3] Dragesco (1968) (*P. coronatum*; *P. balli*), [4] Foissner et al. (1994), [5] Kent (1881), [6] Small and Lynn (1901) (*P. balli*), [7] Song (2000), [8] Dragesco and Dragesco-Kernéis (1986), [9] Corliss and Snyder (1986), [10] Agatha et al. (1993)

^a^Data measured based on drawings or photomicrographs

Based on the presence of approximately 30–60 somatic kineties, a single spherical or ellipsoidal macronucleus, posterior end of M2a (hook-like), the position of the anterior end of the V-shaped M2b slightly above or at the same level as the posterior end of M2a, and the three-rowed membranelle 3, two *Pleuronema* species should be compared with *P. foissneri* sp. nov., namely *P. coronatum* Kent, 1881, and *P. salmastra* Dragesco & Dragesco-Kernéis, 1986 (Fig. 8; Table 3).

*Pleuronema foissneri* sp. nov. can be distinguished from *P. coronatum* mainly in body shape (anterior end slightly narrowed, right ventrolateral side convex in the former vs. anterior end rounded, right ventrolateral side straight in the latter) and the longer M1 relative to the body length (0.14–0.21 vs. 0.08–0.11 in *P. coronatum*) (Borror 1963; Chorik 1968; Dragesco 1968; Foissner et al. 1994; Kent 1881; Small and Lynn 1985; Song 2000).

*Pleuronema foissneri* sp. nov. resembles *P. salmastra* in the slightly narrowed anterior end and in the body size after silver staining. The former, however, differs from the latter by having fewer somatic kineties (32–40 vs. 43–63 in *P. salmastra*), fewer preoral kineties (four to eight, five on average vs. six to ten, eight on average in *P. salmastra*), and a longer M1 relative to the body length (0.14–0.21 vs. 0.09–0.10 in *P. salmastra*) (Dragesco and Dragesco-Kernéis 1986).

Like *Pleuronema foissneri* sp. nov., *P. glaciale* and *P. arctica* both have a narrowed anterior end. However, they can be easily separated from *P. foissneri* sp. nov. by their larger body size (longer than 120 μm after silver staining vs. 60–90 μm in *P. foissneri* sp. nov.) and in having more somatic kineties (44–58 and 39–61, respectively, vs. 32–40 in *P. foissneri* sp. nov.). Furthermore, in *P. glaciale*, the length of M1 is about half (vs. one-third in *P. foissneri* sp. nov.) of M2a length, and the M3 is double-rowed (vs. three-rowed in *P. foissneri* sp. nov.) (Agatha et al. 1993; Corliss and Snyder 1986). For further details of comparisons between *P. foissneri* sp. nov. and its congeners, see Table 3.

### Comments on *Pleuronema parasmalli* sp. nov. (Fig. 8; Table 4)

**Table 4:** Morphological comparison of *Pleuronema parasmalli* sp. nov. with related congeners

Characteristics	*P. parasmalli*	*P. smalli*	*P. paucisaetosum*	*P. setigerum*
Data source	Present work	[1, 2]	[3]	[4–10]
Body size in vivo (μm)	55–85 × 25–35	–	55–85 × 25–55	25–50 × 10–30
Body size after staining (μm)	60–80 × 30–40	40–70 × 25–40	60–82 × 36–58	38–55 × 16–34
Right ventrolateral side in vivo	Basically straight	–	Straight or slightly concave	Straight or slightly concave
Number of caudal cilia	15–18	–	12–15	9–13
Extrusomes	Present	–	Present	Present
Number of SK	26–32	24–36	21–23	12–25
Number of PK	4–6	2–5	4–5	3–6
Posterior end of M2a	Hook-like	Hook-like	Hook-like	Curved to right with a ring-like structure
Shape of Ma	Spherical	Spherical to ellipsoidal	Spherical	Spherical
Diameter of Ma	15–22 μm	–	About 13 μm	About 7–16 μm
Number of Mi	1	2	1–5 (avg. 2)	1–4 ([6])
Habitat	Freshwater	Brackish and marine water	Brackish water	Marine water

*M2a* membranelle 2a, *Ma* macronucleus, *Mi* micronuclei, *PK* preoral kineties, *SK* somatic kineties, – information not available. References: [1] Borror (1972), [2] Dragesco (1968), [3] Pan et al. (2015a), [4] Borror (1963), [5] Calkins (1902), [6] Jung (2021), [7] Kahl (1931), [8] Noland (1937), [9] Pan et al. (2010), [10] Pan et al. (2015b)

Based on its body size and shape (50–90 μm in length, right ventrolateral side straight), number of somatic kineties (20–40), shape of the posterior portion of M2a (hook-like), position of the anterior end of the V-shaped M2b (not above the level of the posterior end of M2a) and three-rowed M3, *Pleuronema parasmalli* sp. nov. should be compared with *P. smalli* Dragesco, 1968, *P. paucisaetosum* Pan et al., 2015, and *P. setigerum* Calkins, 1902 (Fig. 8; Table 4).

*Pleuronema parasmalli* sp. nov. closely resembles *P. smalli* in its small body size after silver staining and similar ciliature pattern. The former, however, can be distinguished from the latter by having a single micronucleus (vs. two in *P. smalli*) and its freshwater habitat (vs. brackish and marine water in *P. smalli*). Morphological information of *P. smalli* in vivo is not available, therefore, a comparison with *P. parasmalli* sp. nov. in vivo cannot be performed (Borror 1972; Dragesco 1968).

*Pleuronema parasmalli* sp. nov. has a similar body size, shape, and oral structure to *P. paucisaetosum*, but the former differs from the latter in having more somatic kineties (26–32 vs. 21–23), a single micronucleus (vs. one to five, two on average), and a freshwater (vs. brackish water) habitat (Pan et al. 2015a).

*Pleuronema setigerum* can be separated from *P. parasmalli* sp. nov. by having a shorter and more slender body (25–50 μm × 10–30 μm in the former vs. 55–85 μm × 25–35 μm in the latter), a ring-like (vs. hook-like in the latter) posterior portion of M2a, fewer caudal cilia (9–13 vs. 15–18), fewer somatic kineties (12–25 vs. 26–32) and a marine (vs. freshwater) habitat (Borror 1963; Calkins 1902; Jung 2021; Kahl 1931; Noland 1937; Pan et al. 2010, 2015b).

### Comments on *Pleuronema parasalmastra* sp. nov. (Fig. 8; Table 5)

**Table 5:** Morphological comparison of *Pleuronema parasalmastra* sp. nov. with related congeners

Characteristics	*P. parasalmastra*	*P. salmastra*	*P. borrori*	*P. coronatum*
Data source	Present work	[1]	[2]	[2] (*P. coronatum*; *P. balli*), [3–8]
Body size in vivo (μm)	90–120 × 40–55	–	–	60–125 × 30–60
Body size after staining (μm)	95–160 × 45–75 (avg. 120 × 57)	50–116 × 22–40	95–122 × 70–77 (avg. 109 × 74)	50–115 × 25–65
Right ventrolateral side in vivo	Straight	Straight	–	Straight
Number of caudal cilia	About 18–20	10^a^	–	10–15
Extrusomes	Present	Present	–	Present
Number of SK	37–43, avg. 40 (*n* = 15)	43–63, avg. 53 (*n* = 20)	41–46, avg. 43	35–48
Number of PK	4–6, usually 6	6–10, avg. 7.6 (*n* = 16)	2–6	3–7
Cell apex to anterior end of M1/body length	0.16–0.25, avg. 0.21 (*n* = 25)	0.08^a^–0.18^a^	0.21^a^	0.09^a^–0.21^a^
Length ratio of oral/body	0.57–0.74	0.55^a^–0.81^a^	0.55^a^	0.64^a^–0.75^a^
Mid-portion of M2a	Single-rowed	Single-rowed	–	Usually in zig-zag pattern
Shape of Ma	Ellipsoidal	Spherical to ellipsoidal	–	Spherical
Diameter of Ma	23–35 μm, avg. 29 μm (*n* = 12)	11–18 μm, avg. 13.5 μm	–	About 15–30 μm
Number of Mi	–	1–14, avg. 4.8 (*n* = 114)	–	1–3
Habitat	Brackish water (salinity 10)	Brackish water	–	Freshwater and marine water

*avg.* average, *M2a* membranelle 2a, *Ma* macronucleus, *Mi* micronuclei, *n* number of examined cells, *PK* preoral kineties, *SK* somatic kineties, – information not available. References: [1] Dragesco and Dragesco-Kernéis (1986), [2] Dragesco (1968), [3] Borror (1963), [4] Chorik (1968), [5] Foissner et al. (1994), [6] Kent (1881), [7] Small and Lynn (1901) (*P. balli*), [8] Song (2000)

^a^Data from drawings or photomicrographs

Based on its body shape (right ventrolateral side straight and dorsal side convex), number of somatic kineties (approximately 30–60), shape of macronucleus (spherical to ellipsoidal), shape of the posterior portion of M2a (hook-like), anterior end of the V-shaped M2b (located at approximately the same level as the posterior end of M2a), and three-rowed M3, three species should be compared with *P. parasalmastra* sp. nov., namely *P. salmastra* Dragesco & Dragesco-Kernéis, 1986, *P. borrori* Dragesco, 1968, and *P. coronatum* Kent, 1881 (Fig. 8; Table 5).

*Pleuronema parasalmastra* sp. nov. can be distinguished from *P. salmastra* by having a larger body size in silver-stained specimens (95–160 μm × 45–77 μm vs. 50–116 μm × 22–40 μm in *P. salmastra*), fewer somatic kineties (37–43, 40 on average vs. 43–63, 53 on average in *P. salmastra*) and fewer preoral kineties (4–6 vs. 6–10 in *P. salmastra*). Furthermore, the distance between the cell apex and the anterior end of M1 occupies a larger portion of the body length in *P. parasalmastra* sp. nov. (0.16–0.25 of body length) than that in *P. salmastra* (0.08–0.18 of body length, data inferred from the drawings of silver-stained specimens) (Dragesco and Dragesco-Kernéis 1986).

*Pleuronema borrori* was established by Dragesco (1968) based only on silver-stained specimens. This species can be characterized by its large body size with a relatively small buccal field. There have been no redescriptions of *P. borrori*. *Pleuronema parasalmastra* sp. nov. resembles *P. borrori* in the large body size with a small and posteriorly positioned buccal field, but it differs from *P. borrori* by having fewer somatic kineties (37–43 vs. 41–46 in *P. borrori*) and a relatively slender body after silver staining (45–75 μm, 57 μm on average in width vs. 70–77 μm, 74 μm on average in *P. borrori*). In addition, the buccal field in *P. parasalmastra* sp. nov. occupies a larger portion of the body length (0.57–0.74 vs. 0.55 in *P. borrori*, inferred from the drawing of a silver-stained cell) (Dragesco 1968).

When compared with *Pleuronema coronatum*, *P. parasalmastra* sp. nov. has a larger body size in silver-stained specimens (95–160 μm in length vs. 50–115 μm in *P. coronatum*) and a more posteriorly positioned buccal field (distance between cell apex to anterior end of M1 in *P. parasalmastra* sp. nov. is 0.16–0.25 of body length vs. 0.09–0.21 in *P. coronatum*). In addition, the mid-portion of M2a is clearly single-rowed in *P. parasalmastra* sp. nov., whereas it has a zig–zag pattern in *P. coronatum* (Borror 1963; Chorik 1968; Dragesco 1968; Foissner et al. 1994; Kent 1881; Small and Lynn 1985; Song and Wilbert 2000).

### Comments on *Pleuronema paraorientale* sp. nov. (Fig. 8; Table 6)

**Table 6:** Morphological comparison of *Pleuronema paraorientale* sp. nov. with related congeners

Characteristics	*P. paraorientale*	*P. orientale*	*P. puytoraci*
Data source	Present work	[1]	[2, 3]
Body size in vivo (μm)	95–115 × 55–70	95–135 × 50–85	70–120 × 45–70
Body size after staining (μm)	115–150 × 60–95	97–133 × 51–84	70–120 × 45–82
Oral length/body length	0.61–0.77 (avg. 0.69)	0.79^a^	0.72^a^ ([3])
Right ventrolateral side in vivo	Convex	Convex	Convex
Extrusomes	Present	Present	Present
Number of SK	52–62 (avg. 56)	42–50 (avg. 47)	28–29 (avg. 28)
Number of PK	3–5	2–3	1–3
Length ratio of M1/M2a	0.19–0.25 (avg. 0.23)	0.21^a^	0.22^a^–0.27^a^ ([2])
Length of M1/body length	0.08–0.13 (avg. 0.10)	0.11^a^	0.07^a^ ([3])
Mid-portion of M2a	Zig-zag pattern	Zig-zag pattern	Zig-zag pattern^a^
Posterior end of M2a	Hook-like	Hook-like	Hook-like
Position of anterior end of M2b	Significantly above posterior end of M2a	Significantly above posterior end of M2a	Same level ([2]) or significantly above posterior end of M2a ([3])
Shape of Ma	Ellipsoidal	Spherical	Spherical to ellipsoidal
Length of Ma	25–45 μm	25 μm	–
Number of Mi	2–10 (avg. 4)	1–3 (avg. 1)	A few ([2])
Diameter of Mi	3–6 μm	–	–
Distance from anterior of M1 to cell apex/body length	0.07–0.16 (avg. 0.12)	0.06^a^	0.08^a^ ([3])
Habitat	Brackish water (salinity 12)	Brackish water (salinity 6.8)	Marine water

*avg.* average, *M1* membranelle 1, *M2a* membranelle 2a, *M2b* membranelle 2b, *Ma* macronucleus, *M*i micronuclei, *PK* preoral kineties, *SK* somatic kineties, *–* information not available. References: [1] Pan et al. (2015a), [2] Groliere and Detcheva (1974), [3] Pan et al. (2011)

^a^Information from drawings or photomicrographs

Based on its body size in vivo (approximately 100–150 μm in length), body shape (right ventrolateral and dorsal sides convex), number of somatic kineties (approximately 30–60), shape of macronucleus (spherical to ellipsoidal), posterior portion of M2a (hook-like), anterior end of the V-shaped M2b (significantly above the posterior end of M2a), and three-rowed M3, there are two species that should be compared with *Pleuronema paraorientale* sp. nov., namely *P. orientale* Pan et al., 2015 and *P. puytoraci* Groliere & Detcheva, 1974 (Fig. 8; Table 6).

*Pleuronema paraorientale* sp. nov. most closely resembles *P. orientale* in its body size, shape, and oral structure. The former, however, is different from the latter mainly by having more somatic kineties (52–62 vs. 42–50 in *P. orientale*), more preoral kineties (3–5 vs. 2–3 in *P. orientale*) and more micronuclei (2–10, four on average vs. 1–3, one on average in *P. orientale*) (Pan et al. 2015a).

*Pleuronema paraorientale* sp. nov. mainly differs from *P. puytoraci* by having more somatic kineties (52–62 vs. 28–29 in *P. puytoraci*) and more preoral kineties (3–5 vs. 1–3 in *P. puytoraci*) (Groliere and Detcheva 1974; Pan et al. 2011).

### Phylogenetic analyses

#### Phylogenetic relationships between *Pleuronema* and *Schizocalyptra*

Studies on the molecular phylogeny of *Pleuronema* began with the sequencing of the large subunit rRNA gene of *P. marinum* (Baroin-Tourancheau et al. 1992). Then the SSU rRNA gene and internal transcribed spacer 2 region data of *P. coronatum* were added and phylogenetic analyses showed that *Pleuronema* was closely related to *Schizocalyptra* and *Cyclidium* (Lynn and Strüder-Kypke 2005; Miao et al. 2008).

According to the phylogenetic analysis in Yi et al. (2009), the genus *Pleuronema* is polyphyletic since two *Schizocalyptra* sequences nest within it forming a fully supported clade. The polyphyly of *Pleuronema* caused by *Schizocalyptra* was subsequently confirmed in most phylogenetic studies based on the SSU rRNA gene (Antipa et al. 2016, 2020; Gao et al. 2012, 2013, 2017; Pan et al. 2010, 2015a, b, 2016, 2020). However, in phylogenetic analyses based on nuclear or mitochondrial data, the genus *Schizocalyptra* falls outside of *Pleuronema* (Gao et al. 2014; Lu et al. 2021; Zhang et al. 2019). Consistent with previous studies based on SSU rRNA gene sequence data, our ML tree shows that *Schizocalyptra* sequences nest within *Pleuronema* (Fig. 6). However, the support values are very low and the phylogenetic position of *Schizocalyptra* is unstable, which may be due to the inclusion of insufficient taxa in the analyses. Considering the low support values and inconsistency of the phylogenetic position of *Schizocalyptra* based on previous and present studies, the relationship between *Pleuronema* and *Schizocalyptra* is still uncertain, and more morphological and molecular data are needed to further clarify their positions.

#### Phylogenetic relationships between the families Peniculistomatidae and Pleuronematidae

Based on SSU rRNA gene data, Antipa et al. (2016) was the first to reveal that the monophyletic family Peniculistomatidae falls within the *Pleuronema* spp., resulting in the polyphyly of Pleuronematidae. In subsequent studies, the polyphyly of Pleuronematidae caused by Peniculistomatidae was verified (Antipa et al. 2020; Lu et al. 2021; Zhang et al. 2019). In the present study, the family Peniculistomatidae nests within the Pleuronematidae in both the ML and BI trees, albeit with low support, supporting previous finding.

*Peniculistoma* and *Mytilophilus* are two endocommensal genera of Peniculistomatidae. Both are characterized by an irregular oval-shaped outline when viewed from the lateral aspect, the cytostome lying at the bottom of a deeply concaved depression, and having numerous somatic kineties, which clearly differentiates these genera from *Pleuronema* (Antipa and Dolan 1985; Antipa et al. 2016; Dolan and Antipa 1985; Fenchel 1965). The oral structures (a long and prominent paroral membrane and three membranelles, with M2 bipartite) and stomatogenesis of *Peniculistoma* and *Mytilophilus*, however, are generally similar with those in *Pleuronema* (Antipa and Dolan 1985; Dolan and Antipa 1985; Fenchel 1965). Furthermore, their phylogenetic positions in the SSU rRNA gene tree reflect the morphological similarities between the families Peniculistomatidae and Pleuronematidae (Fig. 6).

#### Phylogeny of the family Histiobalantiidae

In the phylogenetic analyses by Foissner et al. (2009) (when Histiobalantiidae sequences were first used in molecular phylogeny) and by Antipa et al. (2016, 2020), Histiobalantiidae clustered with *Schizocalyptra*, which was nested within the Pleuronematidae, whereas in other phylogenetic analyses, including the present study (Fig. 6), Histiobalantiidae is invariably placed outside the Pleuronematidae with full support (Antipa et al. 2016; Gao et al. 2012, 2013, 2014, 2017; Lu et al. 2021; Pan et al. 2010, 2015a, b, 2016, 2020; Zhang et al. 2019). In consideration of the insufficient sampling of *Pleuronema* sequences in Foissner et al. (2009) and the relatively low support for the position of *Schizocalyptra* in Antipa et al. (2016, 2020), it is more credible that the phylogenetic position of Histiobalantiidae is outside the Pleuronematidae + Peniculistomatidae clade.

## Materials and Methods

### Sampling and cultivation

Four *Pleuronema* species were collected at Shenzhen, China. *Pleuronema foissneri* sp. nov. was collected from a mangrove wetland in Shenzhen Bay Park (22°31′19.8′′ N; 114°0′11.1′′ E) on 4th April, 2016, with a water temperature of approximately 20 °C and salinity of approximately 8. *Pleuronema parasmalli* sp. nov. was collected from a shallow freshwater pond (the pond was approximately 15 cm deep) at Dameisha sandy beach (22°35′12.7′′ N; 114°18′14.9′′ E) on 17th November, 2016, with a water temperature of approximately 24 °C. *Pleuronema parasalmastra* sp. nov. was isolated from Yelin Sand Beach (22°31′19.5′′ N; 113°59′17.2′′ E) on 17th December, 2015, with a water temperature of approximately 10 °C and a salinity of approximately 10. *Pleuronema paraorientale* sp. nov. was collected from a mangrove wetland on the west coast of Shenzhen Bay (22°30′8.2′′ N; 113°57′10.7′′ E) on 6th January, 2017, where the water temperature was approximately 24 °C and the salinity was approximately 12.

For *Pleuronema foissneri* sp. nov. and *P. paraorientale* sp. nov., water samples were collected from naturally formed small puddles during ebb tide. An approximately 200 ml water sample with wilted leaves and sediment was placed into a 400 ml sampling bottle using bottle caps, and stirring was avoided. For *P. parasmalli* sp. nov., an approximately 200 ml volume of well-stirred freshwater sample with humus was placed into a 400 ml bottle. For *P. parasalmastra* sp. nov., several holes were excavated in the sand to a depth of approximately 10 cm. After water gradually seeped into the holes, water and sand at the bottom of the holes were mixed and collected. An approximately 200 ml water sample was placed into a 400 ml bottle. In all cases, samples were transferred to the laboratory and stored at room temperature (~ 25 °C).

After gently mixing the samples in the bottles, water with sediment of each sample was poured into five 90 mm Petri dishes to establish the initial cultures at ~ 25 °C. No food source was added to these Petri dishes. Three to five cells from the initial cultures were then isolated with micropipettes and transferred into a 35 mm disposable Petri dish with 0.22 µm-filtered water in situ for pure cultivation. Rice grains were added to promote the growth of bacterial food for the ciliates.

### Morphological studies

Living cells were isolated with micropipettes and observed using bright-field and differential interference contrast microscopy at × 100–1000 magnification. The protargol staining method was used to reveal the ciliature and nuclear apparatus (Wilbert 1975). Counts and measurements were performed according to Bai et al. (2020). Drawings of living cells were produced according to Wu et al. (2020). Drawings of silver-stained cells were made using Adobe Photoshop based on photomicrographs of the holotype specimen of each species. Terminology and systematics follow Pan et al. (2016) and Gao et al. (2016), respectively.

### DNA extraction, PCR amplification, and sequencing

Six to ten cells of each species were selected from pure cultures and washed five times with filtered in situ water (0.22 µm, Millex-GP filter unit) to exclude contamination. For each species, cells were then distributed in three Eppendorf tubes (Axygen, USA) with one, three, and multiple individuals. Genomic DNA was extracted using the DNeasy Blood & Tissue kit (Qiagen, Germany) following the optimized manufacturer’s protocol, modified such that 1/4 of the suggested volume was used for each solution. The primers 18S-F, 18S-R (Medlin et al. 1988) and 82F (Jerome et al. 1996) were used for PCR amplifications of the SSU rRNA gene. To minimize the errors caused by PCR, Q5 Hot Start High-Fidelity 2 × Master Mix (New England BioLabs, USA) was used as DNA polymerase. The PCR parameters were utilized according to Jiang et al. (2021). After amplification, PCR products were sequenced bidirectionally by the Tsingke Biological Technology Company (Qingdao, China).

### Phylogenetic analyses

The SSU rRNA gene sequences of the four *Pleuronema* species in the present work were combined with 64 sequences of related taxa downloaded from GenBank, forming the dataset for phylogenetic analyses (for accession numbers, see Fig. 6). Ten sequences from Philasterida were selected as the outgroup. All sequences were aligned using the MAFFT algorithm on GUIDANCE2 Server (http://guidance.tau.ac.il) with default parameters (Penn et al. 2010; Sela et al. 2015). The resulting alignment was manually edited using the program BioEdit version 7.0.5.2 (Hall 1999), and both ends of the alignment were trimmed. The final alignment, including 1802 positions, was used to construct the phylogenetic trees.

Maximum likelihood (ML) analysis with 1000 bootstrap replicates was performed using RAxML-HPC2 on XSEDE 8.2.10 (Stamatakis 2014) under the GTRGAMMA model at CIPRES Science Gateway (http://www.phylo.org/sub_sections/portal) (Miller et al. 2010). Bayesian inference (BI) was performed with MrBayes 3.2.6 on XSEDE 3.2.6 (Ronquist and Huelsenbeck 2003) at the CIPRES Science Gateway with the best-fit model GTR + I + G, selected under the Akaike Information Criterion using MrModeltest 2 (Nylander 2004). Markov chain Monte Carlo (MCMC) simulations were run with two sets of four chains for 10,000,000 generations at a sampling frequency of 100 and a burn-in of 10,000 trees (10%). Convergence of the MCMC analyses was confirmed in that the average standard deviation of split frequencies was well below 0.01. All remaining trees were used to calculate posterior probabilities (PP) using a 50% majority rule consensus. Tree topologies were visualized using SeaView version 4 (Gouy et al. 2010). The SSU rRNA gene comparison of all *Pleuronema* sequences in the present work was performed by BioEdit version 7.0.5.2 (Hall 1999). The alignment length had 1623 positions after trimming both ends.

## Supplementary Information

Below is the link to the electronic supplementary material.

Supplementary file1 (DOC 105 KB)

## Data Availability

The datasets presented in this study can be found in online repositories. The names of the repository/repositories and accession number(s) can be found at: https://www.ncbi.nlm.nih.gov/genbank/ (OL654416, OL654417, OL654418, and OL654419).
