# Supplementary material for: Integrative studies on the taxonomy and molecular phylogeny of four new Pleuronema species (Protozoa, Ciliophora, Scuticociliatia)
Source: Mar Life Sci Technol. 2022 May 12;4(2):179–200. doi: 10.1007/s42995-022-00130-5 (PMC10077198; doi:10.1007/s42995-022-00130-5)
Supplement: Supplementary file 1 — Supplementary file1 (DOC 105 KB) [file 42995_2022_130_MOESM1_ESM.doc]

**Table S1.** Comparison of SSU rRNA gene sequences within the genus *Pleuronema*.

|  | **Sequences** | *1* | *2* | *3* | *4* | *5* | ***6*** | ***7*** | ***8*** | ***9*** | *10* | *11* | *12* | *13* | *14* | *15* | *16* | *17* | *18* | *19* | *20* | *21* | *22* | *23* | *24* |
| --- | --- | --- | --- | --- | --- | --- | --- | --- | --- | --- | --- | --- | --- | --- | --- | --- | --- | --- | --- | --- | --- | --- | --- | --- | --- |
| *1* | *Pleuronema coronatum* AY103188 | - | 0 | 4 | 97 | 99 | 98 | 96 | 97 | 123 | 122 | 85 | 82 | 58 | 63 | 60 | 57 | 58 | 94 | 98 | 97 | 124 | 121 | 133 | 86 |
| *2* | *Pleuronema coronatum* HM140396 | 100 | - | 4 | 97 | 99 | 98 | 96 | 97 | 123 | 122 | 85 | 82 | 58 | 63 | 60 | 57 | 58 | 94 | 98 | 97 | 124 | 121 | 133 | 86 |
| *3* | *Pleuronema coronatum* JX310018 | 99.7 | 99.7 | - | 93 | 95 | 94 | 92 | 93 | 120 | 118 | 81 | 78 | 54 | 59 | 56 | 53 | 54 | 90 | 94 | 93 | 122 | 119 | 129 | 82 |
| *4* | *Pleuronema puytoraci* KF840520 | 93.9 | 93.9 | 94.2 | - | 2 | 19 | 43 | 51 | 93 | 119 | 109 | 102 | 89 | 95 | 92 | 92 | 90 | 123 | 127 | 123 | 150 | 150 | 147 | 118 |
| *5* | *Pleuronema orientale* KF206429 | 93.8 | 93.8 | 94.0 | 99.8 | - | 21 | 45 | 53 | 95 | 121 | 111 | 104 | 91 | 97 | 94 | 94 | 92 | 125 | 129 | 125 | 150 | 150 | 147 | 120 |
| ***6*** | ***Pleuronema paraorientale* sp. nov. OL654419** | 93.9 | 93.9 | 94.1 | 98.8 | 98.6 | - | 44 | 63 | 102 | 125 | 111 | 104 | 91 | 100 | 97 | 97 | 93 | 125 | 129 | 124 | 154 | 154 | 147 | 120 |
| ***7*** | ***Pleuronema foissneri* sp. nov. OL654416** | 94.0 | 94.0 | 94.2 | 97.3 | 97.2 | 97.2 | - | 62 | 102 | 121 | 111 | 104 | 90 | 96 | 93 | 91 | 94 | 122 | 126 | 121 | 151 | 151 | 146 | 115 |
| ***8*** | ***Pleuronema parasalmastra* sp. nov. OL654418** | 93.9 | 93.9 | 94.2 | 96.8 | 96.7 | 96.0 | 96.1 | - | 82 | 114 | 112 | 105 | 85 | 93 | 90 | 86 | 88 | 120 | 124 | 124 | 151 | 150 | 147 | 113 |
| ***9*** | ***Pleuronema parasmalli* sp. nov. OL654417** | 92.3 | 92.3 | 92.5 | 94.2 | 94.1 | 93.6 | 93.6 | 94.9 | - | 135 | 135 | 128 | 107 | 122 | 119 | 118 | 116 | 151 | 155 | 145 | 168 | 167 | 164 | 129 |
| *10* | *Pleuronema setigerum* FJ848874 | 92.4 | 92.4 | 92.6 | 92.5 | 92.4 | 92.2 | 92.4 | 92.9 | 91.6 | - | 146 | 142 | 129 | 138 | 135 | 128 | 131 | 147 | 151 | 144 | 180 | 179 | 177 | 156 |
| *11* | *Pleuronema coronatum* JX310014 | 94.7 | 94.7 | 94.9 | 93.2 | 93.1 | 93.1 | 93.1 | 93.0 | 91.6 | 90.9 | - | 9 | 57 | 89 | 86 | 90 | 81 | 123 | 127 | 124 | 148 | 146 | 160 | 118 |
| *12* | *Pleuronema elegans* KF840518 | 94.9 | 94.9 | 95.1 | 93.6 | 93.5 | 93.5 | 93.5 | 93.4 | 92.0 | 91.1 | 99.4 | - | 55 | 86 | 83 | 86 | 79 | 118 | 122 | 121 | 144 | 142 | 156 | 112 |
| *13* | *Pleuronema binucleatum* KT033424 | 96.3 | 96.3 | 96.6 | 94.4 | 94.3 | 94.3 | 94.4 | 94.7 | 93.3 | 91.9 | 96.4 | 96.5 | - | 54 | 51 | 54 | 50 | 94 | 98 | 94 | 122 | 120 | 130 | 93 |
| *14* | *Pleuronema grolierei* KF840519 | 96.0 | 96.0 | 96.3 | 94.0 | 93.9 | 93.7 | 94.0 | 94.2 | 92.4 | 91.4 | 94.4 | 94.6 | 96.6 | - | 3 | 20 | 23 | 95 | 99 | 98 | 118 | 116 | 126 | 89 |
| *15* | *Pleuronema setigerum* JX310015 | 96.2 | 96.2 | 96.5 | 94.2 | 94.1 | 93.9 | 94.2 | 94.4 | 92.6 | 91.6 | 94.6 | 94.8 | 96.8 | 99.8 | - | 17 | 20 | 92 | 96 | 95 | 115 | 113 | 123 | 86 |
| *16* | *Pleuronema paucisaetosum* KF206430 | 96.4 | 96.4 | 96.7 | 94.2 | 94.1 | 93.9 | 94.3 | 94.6 | 92.6 | 92.0 | 94.4 | 94.6 | 96.6 | 98.7 | 98.9 | - | 24 | 96 | 100 | 101 | 120 | 119 | 125 | 88 |
| *17* | *Pleuronema* cf. *setigerum* FJ848875 | 96.3 | 96.3 | 96.6 | 94.4 | 94.2 | 94.2 | 94.1 | 94.5 | 92.8 | 91.8 | 94.9 | 95.0 | 96.8 | 98.5 | 98.7 | 98.5 | - | 93 | 97 | 93 | 116 | 116 | 128 | 87 |
| *18* | *Pleuronema marinum* KF206428 | 94.1 | 94.1 | 94.3 | 92.3 | 92.2 | 92.2 | 92.4 | 92.5 | 90.6 | 90.8 | 92.3 | 92.6 | 94.1 | 94.1 | 94.2 | 94.0 | 94.2 | - | 14 | 47 | 135 | 132 | 138 | 104 |
| *19* | *Pleuronema* sp. FJ848876 | 93.9 | 93.9 | 94.1 | 92.1 | 91.9 | 91.9 | 92.1 | 92.2 | 90.3 | 90.6 | 92.1 | 92.4 | 93.9 | 93.8 | 94.0 | 93.7 | 93.9 | 99.1 | - | 51 | 135 | 132 | 138 | 104 |
| *20* | *Pleuronema sinica* EF486864 | 93.9 | 93.9 | 94.2 | 92.3 | 92.2 | 92.2 | 92.4 | 92.2 | 91.0 | 91.0 | 92.3 | 92.4 | 94.1 | 93.9 | 94.1 | 93.7 | 94.2 | 97.0 | 96.8 | - | 136 | 133 | 143 | 102 |
| *21* | *Pleuronema parawiackowskii* KT033423 | 92.3 | 92.3 | 92.4 | 90.7 | 90.7 | 90.4 | 90.6 | 90.6 | 89.6 | 88.8 | 90.8 | 91.0 | 92.4 | 92.6 | 92.8 | 92.5 | 92.8 | 91.6 | 91.6 | 91.5 | - | 9 | 81 | 90 |
| *22* | *Pleuronema wiackowskii* JX310016 | 92.4 | 92.4 | 92.6 | 90.7 | 90.7 | 90.4 | 90.6 | 90.7 | 89.6 | 88.8 | 90.9 | 91.2 | 92.5 | 92.8 | 92.9 | 92.6 | 92.8 | 91.8 | 91.8 | 91.7 | 99.4 | - | 77 | 88 |
| *23* | *Pleuronema czapikae* EF486863 | 91.7 | 91.7 | 91.9 | 90.8 | 90.8 | 90.8 | 90.9 | 90.8 | 89.8 | 88.9 | 90.0 | 90.3 | 91.9 | 92.1 | 92.3 | 92.2 | 92.0 | 91.4 | 91.4 | 91.1 | 94.9 | 95.2 | - | 100 |
| *24* | *Pleuronema* sp. JX310017 | 94.6 | 94.6 | 94.9 | 92.6 | 92.5 | 92.5 | 92.8 | 92.9 | 92.0 | 90.3 | 92.6 | 93.0 | 94.2 | 94.4 | 94.6 | 94.5 | 94.5 | 93.5 | 93.5 | 93.6 | 94.4 | 94.5 | 93.7 | - |

Sequences are ordered according to the SSU rRNA gene tree. The four newly submitted sequences in the present work are in bold. The number of unmatched nucleotides is shown above the diagonal, while the percentage of sequence identity (%) is below the diagonal. Numbers shaded in the same block indicate the corresponding sequences belong to a common clade in the SSU rRNA gene tree.
